# Effects of prenatal photobiomodulation treatment on neonatal hypoxic ischemia in rat offspring

**DOI:** 10.7150/thno.49672

**Published:** 2021-01-01

**Authors:** Luodan Yang, Yan Dong, Chongyun Wu, Hannah Youngblood, Yong Li, Xuemei Zong, Lei Li, Tongda Xu, Quanguang Zhang

**Affiliations:** 1 Department of Neuroscience and Regenerative Medicine, Medical College of Georgia, Augusta University, 1120 15th Street, Augusta, GA 30912, USA.; 2 Department of Cellular Biology and Anatomy, Medical College of Georgia, Augusta University, 1120 15th Street, Augusta, GA 30912, USA.

**Keywords:** Prenatal photobiomodulation, Neonatal hypoxic-ischemic encephalopathy, Mitochondria, Neuroinflammation, Oxidative stress

## Abstract

Neonatal hypoxic-ischemic (HI) injury is a severe complication often leading to neonatal death and long-term neurobehavioral deficits in children. Currently, the only treatment option available for neonatal HI injury is therapeutic hypothermia. However, the necessary specialized equipment, possible adverse side effects, and limited effectiveness of this therapy creates an urgent need for the development of new HI treatment methods. Photobiomodulation (PBM) has been shown to be neuroprotective against multiple brain disorders in animal models, as well as limited human studies. However, the effects of PBM treatment on neonatal HI injury remain unclear.

**Methods:** Two-minutes PBM (808 nm continuous wave laser, 8 mW/cm^2^ on neonatal brain) was applied three times weekly on the abdomen of pregnant rats from gestation day 1 (GD1) to GD21. After neonatal right common carotid artery ligation, cortex- and hippocampus-related behavioral deficits due to HI insult were measured using a battery of behavioral tests. The effects of HI insult and PBM pretreatment on infarct size; synaptic, dendritic, and white matter damage; neuronal degeneration; apoptosis; mitochondrial function; mitochondrial fragmentation; oxidative stress; and gliosis were then assessed.

**Results:** Prenatal PBM treatment significantly improved the survival rate of neonatal rats and decreased infarct size after HI insult. Behavioral tests revealed that prenatal PBM treatment significantly alleviated cortex-related motor deficits and hippocampus-related memory and learning dysfunction. In addition, mitochondrial function and integrity were protected in HI animals treated with PBM. Additional studies revealed that prenatal PBM treatment significantly alleviated HI-induced neuroinflammation, oxidative stress, and myeloid cell/astrocyte activation.

**Conclusion:** Prenatal PBM treatment exerts neuroprotective effects on neonatal HI rats. Underlying mechanisms for this neuroprotection may include preservation of mitochondrial function, reduction of inflammation, and decreased oxidative stress. Our findings support the possible use of PBM treatment in high-risk pregnancies to alleviate or prevent HI-induced brain injury in the perinatal period.

## Introduction

Neonatal hypoxic-ischemic encephalopathy (HIE) is a type of brain damage which occurs during the perinatal period due to impaired cerebral blood flow and oxygen deprivation 1, 2. It is the most common cause of death in human neonates with a relatively high incidence rate (~0.4%) and a more than 20% infant mortality rate worldwide 1, 3. Among the patients that survive neonatal HIE, 5-10% suffer from life-long motor deficits while 20-50% experience sensory or cognitive abnormalities 1, 3-5. Although progress in intensive care and assisted respiration technology has increased HIE survival rate, surviving individuals and their families still face life-altering medical difficulties 6, 7.

Currently, the only treatment option available is therapeutic hypothermia 8-11. However, therapeutic hypothermia is only effective if applied during a narrow window of time 8-11. Even if the treatment is applied successfully during that critical period, therapeutic hypothermia has limited efficacy and comes with potential adverse cardiovascular effects 2, 12. Therefore, a critical need exists for a more practical and effective therapy for neonatal HIE.

Neurons are very sensitive to oxygen deprivation because of the high-energy demands of synaptic transmission 1, 13. Because of their role in energy production, mitochondria are a major target of HI injury 14. During HI insults, mitochondrial dysfunction leads not only to decreased ATP generation, but also to excess production of ROS and release of pro-apoptotic proteins, all of which contribute to neuronal damage 1, 15-17. Furthermore, HI-induced mitochondrial dysfunction has been shown to promote the production of inflammatory cytokines and to exacerbate neuroinflammation 18, 19. Recent studies report that mitochondrial fragmentation aggravates neuroinflammation by triggering astrocyte conversion from a neuroprotective A2 phenotype to a pro-inflammatory A1 state 20, 21. Conversely, restoration of mitochondrial function has been shown to reprogram the pro-inflammatory phenotype of myeloid cell (M1) to its anti-inflammatory phenotype (M2) 22, 23. Because oxidative stress, glial activation, and neuroinflammation play significant roles in ischemic brain injury, and mitochondrial function is related to these processes, mitochondria should be considered as possible therapeutic targets for HI brain injury 24-27.

PBM, also known as low-level laser therapy, is an emerging method targeting the mitochondria 28. In the past few years, PBM has evidenced protective effects against mitochondrial dysfunction and neuronal death in multiple brain injury models 26, 29-31. One of our previous studies demonstrated that PBM preconditioning could prevent cognitive impairment in an HI animal model. Further in-depth study about the mechanism of action is needed 1.

Unilateral ligation of the carotid artery followed by hypoxic exposure is a widely used rodent model of neonatal HI 2, 26, 32. Sensorimotor deficits 33, 34 and learning and memory deficits 1, 32 have been reported in juvenile and adult animals subjected to an HI insult during the perinatal period. A number of tests have been developed to assess both short- and long-term functional deficits. Righting reflex has been reported as an effective method to measure short-term functional deficits 35. Behavioral tests for measurement of somatosensory function, motor coordination, asymmetry in forelimb use, and memory functions have been widely used for testing long-term functional deficits after HI insult 36-38. Therefore, the current study was designed to investigate the effects of prenatal PBM treatment on neonatal HI rats using a variety of these techniques.

## Methods

### Animals and experimental design

**Figure 1:**
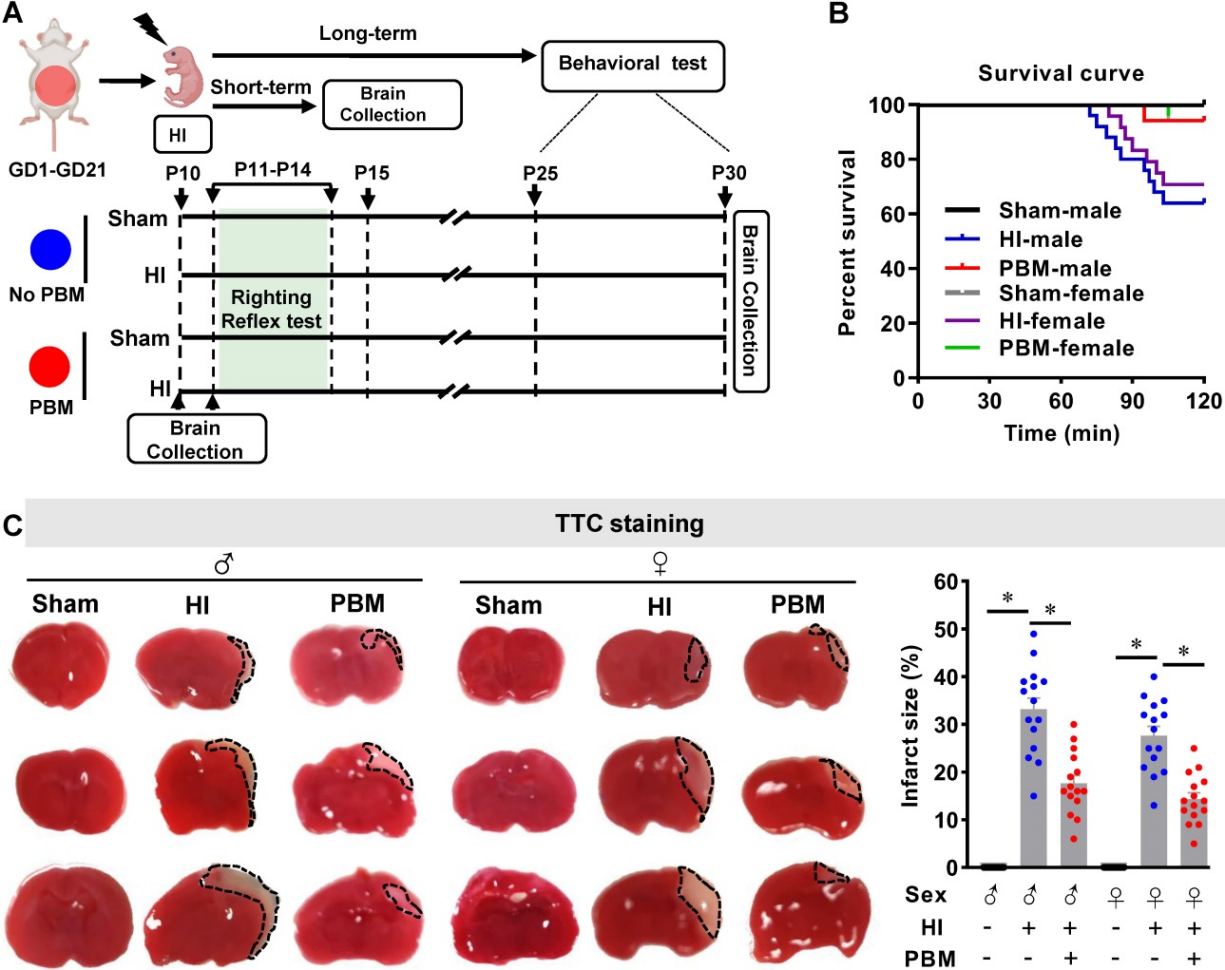
** PBM improves survival rate and decreases infarct size. (A)** Prenatal PBM treatment was applied on the abdomen of pregnant females from GD1 to GD21 three times weekly. After natural delivery, neonates were divided into four groups: a sham group without prenatal PBM treatment (sham), an HI group without prenatal PBM treatment (HI), a sham group with prenatal PBM treatment (PBM+sham), and an HI group with prenatal PBM treatment (PBM). The HI insult was induced on P10. Righting reflex tests were performed from P11 to P14. Other behavioral tests were performed from P25 to P30. Rats were sacrificed on P10, P11, P15 and P30, and brains were prepared for further analysis. The blue circle indicates no PBM treatment and the red circle indicates PBM treatment. **(B)** Kaplan-Meier survival curves suggested that PBM was able to improve the survival rate of neonatal HI rats. **(C)** Infarct size was reduced in HI rats treated prenatally with PBM compared to those not treated. Infarct size was quantized by Image J software and expressed as a percentage of the total area of the contralateral hemisphere. All data are expressed as mean ± SEM (n=15-25). * indicates *P* < 0.05. ♂ indicates male; ♀ indicates females.

Eleven-week-old male and female Sprague-Dawley rats (200-250 g body weight, Charles River Laboratories) were used in this study. Animals were maintained in propylene cages with a controlled ambient temperature of 23 ± 2 °C, a humidity kept between 50-60%, and a 12/12 light-dark cycle with ad libitum access to food and water. Females were mated overnight with one male each. The following morning was considered as gestation day 0 (GD0) if a vaginal plug was observed or sperm was found in a vaginal smear. Thirty pregnant animals were randomly divided into two groups: a group treated with PBM (n = 15) and a group not treated with PBM (n = 15). PBM-untreated animals underwent identical procedures except that the laser power was not turned on. For PBM treatment, 2 min laser irradiation was applied three times weekly on the abdomen of pregnant females from GD1 to GD21 using a Diode IR Laser System (808M100, Dragon Lasers) (**Figure. 1A; Table S1**). The laser setup was based on our previous work with some improvements 39. All the animals with PBM treatment were transiently restrained in a transparent DecapiCone (DCL-120, Braintree Scientific, Inc, MA, USA) during sham treatment or PBM treatment and released to their cages immediately after PBM treatment. The laser fiber optic was connected to a focus lens (Imeter 400 um multi model; Multi-mode Coupler with SMA connector; adjustable focus). The distance between the end of lens and rat abdomen was adjusted to 25 cm, resulting in a 5 cm diameter laser spot on the abdomen of pregnant rats. The reading on top of the abdomen, using our animals, was 350 mW/cm^2^. The laser power density was able to propagate through the abdominal tissue and the power density on neonatal brain was measured and adjusted to 8 mW/cm^2^ using the laser power probes of laser meters (#LP1, Sanwa, Japan; #FM33-056, Coherent Inc, USA). PBM treatments were performed between 5:00 p.m. and 7:00 p.m. After natural delivery, neonatal rats were further divided into 4 groups: a sham group without prenatal PBM treatment (Sham), an HI group without prenatal PBM treatment (HI), a sham group with prenatal PBM treatment (PBM+Sham), and an HI group with prenatal PBM treatment (PBM). There were no differences in the behavioral tests and some key molecular tests between normal animals with/without prenatal PBM treatment (**Figure S1-S3**). Therefore, the PBM+Sham group was not included in the other tests. The HI model described below was initiated at postnatal day 10 (P10) 40-42. Brains were collected at P10 for ATP, cytochrome *c* oxidase activity and MitoTracker® Red measurements; P11 for TTC staining; P15 for quantification of short-term effects with western blotting, ELISA, immunofluorescence, and related assay kits. From P25 to P30, a battery of behavioral tests was performed. The investigators conducting behavioral tests, microscopic imaging, histological counting and other evaluations were blinded to the experimental treatment and staining groups. Randomization was applied to assign animal groups and to collect and analyze data. All procedures were approved by the Institutional Animal Care and Use Committee (IACUC) of Augusta University and complied with National Institutes of Health guidelines.

### Hypoxic-ischemic animal model

HI insult was induced as described in our previous work 1, 43. Briefly, neonatal Sprague-Dawley rats (25 ± 1.6 g) were anesthetized followed by right common carotid artery (RECCA) sterile isolation and permanent ligation. Following closure of the wound, the rat pups were returned to their cages for 1.5 h recovery at 37 °C. Then were placed in a hypoxic environment (6% oxygen, 94% nitrogen) at 37 °C for 2 h. Following hypoxic exposure, they recovered at 37 °C for up to 60 min, then they were returned to their respective home cages for future observation. For the sham group, a sham experiment conducted with identical procedures except ligation and hypoxic exposure. No intra-hypoxic, nesting and core temperature changes were found in PBM treatment group.

### Behavioral tests

Animal's behavioral tests were performed from P11 to P14, P25 to P30 as follows:

(a) The adhesive removal test was performed to measure somatosensory deficits as described in our previous studies 26, 27. In brief, two small adhesive strips (0.35 cm × 0.45 cm) were used as stimuli to the animal's front paw, and then the animals were returned to their home cages in the absence of other rats. Time spent on removing the adhesive strips was recorded during a 2-minute test. If the animal could not remove the adhesive strips within 2 min, the recorded time for this test was 120 s.

(b) The cylinder test was adopted to measure asymmetry of contralateral forepaw use as described in our previous work 26, 27. Briefly, the animals were placed in a transparent glass cylinder (height: 15 cm; diameter: 10 cm) and allowed to touch the cylinder freely for 2 min. During this test, the number of contacts with the wall of the cylinder using left or right paws was counted by two experimenters. The percentage of contralateral (left) paw use was calculated using the following formula: Left paw use (%) = contralateral paw use/number of total paw use.

(c) The edge beam test was used to assess motor balance and coordination as previously described by our laboratory 44. The animals were habituated to the balance beam (length: 100 cm; width: 7 cm; height: 100 cm above the ground) the day prior to the testing day. During habituation and testing, the animals were placed at one end of the beam and allowed to cross the beam to return to their home cage. Time taken to cross the beam and the numbers of missteps were recorded and compared between groups.

(d) The ladder dexterity test was widely used to evaluate motor coordination as reported in our recent work 27. The apparatus used for this test consists of two Plexiglas side walls (100 cm long) linked by metal rungs (3 mm diameter). The ladder rungs were placed 30 cm above the ground with irregular intervals (from 1 to 3 cm). During this test, the animals were placed at one end of the ladder and allowed to cross the ladder to their home cages at the other end. The numbers of missteps were recorded and compared between groups.

(e) The grip test, also known as the hanging wire test, was used to test forelimb motor coordination and grip strength 27. A metal wire (55 cm length) was stretched between two vertical stands and suspended 60 cm above the ground. A rat was handled by the tail and guided to grasp the wire with their forepaws. As soon as the rat was properly suspended, the researcher allowed it to hang by itself. Hanging wire score and hanging time during this 2-minute test were recorded. Hanging wire score was determined by a 5-point scale: 0, rat falls off the wire immediately; 1, rat keeps its start position hanging onto the wire by two forepaws; 2, rat attempts to climb onto the metal wire with 2 forepaws hanging on the wire as for 1; 3, rat grasps the wire as for 1 and attempt to climb the wire with one or both hind limbs; 4, rat grasps the metal wire with both forepaws and hind limbs, as well as wrapping its tail around the wire. Three trials with 5-minute intervals were performed for each animal. The highest hanging score and the longest hanging time were recorded.

(f) The righting reflex test was a widely used basic motor coordination test for rats at early stages of development. As described in our previous study, neonatal rats were placed flat on their back and then released 43. Time taken to roll over from their back onto their paws was recorded. This test was performed from P11 to P14 in our study.

(g) The Barnes maze task is a widely accepted test to measure spatial learning and memory 1, 26, 45, 46. In the current study, a platform (100 cm high) with 18 holes was divided into four quadrants with equal areas, and the quadrant with an escape box (height: 12 cm; width: 15 cm; length: 20 cm) located under one of its holes was defined as the target quadrant. During the 3-minute trials on P27-P29, the rats were placed on the platform and allowed to explore freely. The traces of rats and the time spent on finding the target box were recorded by an overhead video camera controlled by ANY-maze video tracking software (Stoelting, Wood Dale, IL, USA). On the probe day (P30), the escape box was removed and the animals were allowed 90 s to find the escape box (probe trials). The time spent in the target quadrant and the number of exploring errors during the probe test were recorded and analyzed.

(h) The novel object recognition (NOR) test was adopted to evaluate recognition memory as described previously by our laboratory 1, 45. The NOR test is based on rats' intrinsic tendency to spend more time exploring a novel object than a familiar object 1. The NOR test was divided into 3 stages. On the first day of the test, the rats received 5 min to freely explore an empty box (height: 40 cm; width: 50 cm; length: 50 cm) for habituation. On the second day (sampling stage), two identical objects were fixed to the floor of the box at an equal distance, and the rats were allowed to explore the two objects for 5 min. On the third day (choice stage), the animals were returned to the box with one familiar object and one novel object. The traces and the time spent exploring each object were recorded using ANY-maze video tracking software (Stoelting). The data were analyzed and compared between groups.

### Brain collection and tissue preparation

Brain collections were performed on P10, P11, P15 and P30 to measure total ATP content and cytochrome c oxidase activity, TTC staining and short-term/long-term molecular changes. As described in our previous study 43, the brains of rats were quickly removed under deep anesthesia followed by perfusion with ice-cold saline. The cortex and hippocampus were micro-dissected from the ipsilateral damaged hemisphere and frozen immediately for protein analysis. Brains used for tissue section were post-fixed with 4% paraformaldehyde followed by cryoprotection with 30% sucrose. Frozen sections (25 µm) for histological analysis were cut using a Leica Rm2155 microtome. Brains used for protein analysis were homogenized as described in our previous protocol 46, 47 using a motor-driven Teflon homogenizer and ice-cold homogenization medium. Total protein fractions, cytosolic protein fractions, and mitochondrial protein fractions were obtained.

### Immunofluorescence staining and confocal microscopy

Immunofluorescence staining and confocal microscopy were performed following the protocol described in a previous study 45. Briefly, after brain slice preparation, 3-5 sections from each animal were selected for confocal microscopy imaging. The coronal slices collected above were permeabilized with 0.4% Triton X-100 for 5 h and blocked with 10% normal donkey serum for 1 h at room temperature. Next, the brain sections were incubated with the appropriate primary antibodies overnight at 4 °C. The following primary antibodies were used: anti-spinophilin, synaptophysin, 8-OHdG, MFN2, Bcl-2 and 4-HNE (Abcam, Cambridge, UK); anti-GFAP, MAP2, S100A10, and C3d (Thermo Fisher Scientific, Waltham, MA, USA); anti-Iba1, NeuN, MBP, p-H2A.X, CD206, Tom20, and CD16/32 (Proteintech, Rosemont, IL, USA); anti-Cle-caspase 3 (Cell Signaling Technology, Danvers, MA, USA); and anti-SOD2 (Novus Biologicals, Littleton, CO, USA). The following day, the brain slices were washed three times using 0.1% Triton X-100 followed by incubation with Alexa Fluor donkey anti-mouse/goat/goat secondary antibodies (594/647/488, Thermo Fisher Scientific) for 1 h at room temperature. After washing, the sections were mounted with DAPI Fluoromount-G® Mounting Medium (SouthernBiotech, Birmingham, AL, USA).

To measure the depolarization mitochondrial membrane potential (MMP), MitoTracker® Red CMXRos (M-7512, Life Technologies, Carlsbad, CA, USA) was used as described in a previous study 48. Brain collection was performed 6 h after HI insult. In brief, 50 ng/mL MitoTracker® Red CMXRos (M-7512, Life Technologies) in 100 μL of saline was administered by intraperitoneal injection 5 min before brain collection. Fresh tissues were used in this measurement.

All fluorescent images were captured by a LSM700 Meta confocal laser scanning microscope (Carl Zeiss, White Plains, NY, USA) and analyzed using ImageJ software (Version 1.49, NIH, Bethesda, MD, USA).

### TTC staining

TTC (2,3,5-triphenyltetrazolium chloride monohydrate) staining was performed to measure infarct volume according to a previous study 49. In brief, brains were collected 24 h post-HIE and sectioned into 2 mm slices using a vibrating blade microtome (LeicaVT1200 S). Afterwards, brain sections were immersed in TTC solution (2%) at 37 °C for 10 min. Image J (NIH) was used to quantify the mean infarct area of each brain section. Infarct volume was calculated following formula: Infarct size % = infarct area / area of contralateral hemisphere × 100%.

### Western blotting analysis

The concentration of total protein collected was determined using a Modified Lowry Protein Assay kit (Pierce, Rockford, IL, USA). As previous described 1, proteins (50 μg) were separated on sodium dodecyl sulfate polyacrylamide gels at 100 V for 40-50 min and transferred onto a polyvinylidene difluoride (PVDF) membrane. The PVDF membranes were then blocked with bovine serum albumin (BSA) for 30 min followed by incubation overnight with the following antibodies: TGF-β, ARG, CD206, CD86, iNOS, CD32, Bcl-2, cleaved-Caspase 3, and β-actin (Proteintech). Membranes were then washed three times and incubated with the appropriate HRP-conjugated secondary antibody (Cell Signaling) for 1 h at room temperature. After washing three times, images were captured by a cold CCD digital imaging system and analyzed by ImageJ software (Version 1.49, NIH). Band densities were normalized to the loading control and expressed as a percentage of the sham group.

### F-Jade C and TUNEL staining

F-jade C and TUNEL staining was performed following our previously published procedures 27. In brief, the collected brain sections were incubated with Fluoro-jade C (Sigma-Aldrich, St. Louis, MO, USA) working solution for 20 min following the manufacturer's protocol. Sections were then washed 5 times with Triton X-100 in PBS and mounted using mounting medium. A Click-iT® Plus TUNEL assay kit (Thermo Fisher Scientific) was used to label apoptotic cells following the manufacturer's protocol. All images were captured by a LSM700 Meta confocal laser scanning microscope (Carl Zeiss).

### Caspase 3 and caspase 9 activity

Caspase 3 and caspase 9 activity in the protein samples from rat cortex and hippocampus were measured using fluorometric substrates as described previously 48. Ac-DEVD-AMC and Ac-LEHD-AMC (AnaSpec, Fremont, CA, USA) were used as the substrates for caspase 3 and caspase 9, respectively. The fluorescence of free AMC was measured using a microplate reader (BioTek, Winooski, VT, USA) at 360 nm excitation/460 nm emission. The values were expressed as a percentage of change compared with the sham group.

### Lipid Peroxidation (MDA) Assay

A lipid peroxidation (MDA) assay kit (ab118970; Abcam, Cambridge, United Kingdom) was used to measure malondialdehyde production in the cortex and hippocampus according to the instructions of the vendor. In brief, 200 µL of protein samples were mixed with 600 µL of TBA reagent followed by incubation at 95 °C for 60 min. After cooling to room temperature in an ice bath for 10 min, 200 µL of supernatant was moved into a 96-well microplate and measured on a microplate reader at 532 nm. Data was analyzed to determine the optical density value (OD).

### Quantification of total ATP content and cytochrome *c* oxidase activity

The ATP content in rat cortex and hippocampus was determined using a kit of ENLITEN® rLuciferase/Luciferin Reagent (FF2021; Promega, Madison, WI, USA) as described in our previous study 1. Briefly, 30 μg total protein samples were mixed with 100 μL reconstituted rL/L reagent buffer composed of D-luciferin, luciferase, Tris-acetate buffer (pH 7.75), EDTA, bovine serum albumin, dithiothreitol, and magnesium acetate. A standard microplate luminometer (Applied Biosystems, Waltham, MA, USA) was used to measure ATP levels.

Cytochrome *c* oxidase activity was measured using mitochondrial fractions with an activity assay kit (ab109911; Abcam) according to our previously published protocol 26, 48. Briefly, mitochondrial fractions were loaded onto a microtiter plate and incubated for 3 h at room temperature. After washing with Solution 1 provided in the kit, Assay Solution was added to the 96-well plate. Cytochrome* c* oxidase activity was then measured using a Benchmark Plus microplate spectrophotometer (Bio-Rad, Hercules, CA, USA) at 550 nm absorbance. All data were expressed as a percentage of the sham group.

### Total antioxidant capacity

An Antioxidant Assay Kit (Cayman Chemical, Ann Arbor, MI, USA) was used to measure the total antioxidant capacity of rat cortex and hippocampus according to the instructions of the vendor and the description in our previous study 46. In brief, protein samples pre-diluted in assay buffer were mixed with chromogen (150 µL) and metmyoglobin (10 µL) as provided in the assay kit. After adding 40 µL hydrogen peroxide working solution, the plates were incubated for 5 min on a shaker. Thereafter, the optical density value (OD) was measured at 750 nm using a spectrophotometer. The antioxidant capacity of each sample was determined using a Trolox standard curve. The total antioxidant capacity was expressed as a percentage change compared to the sham group.

### Protein carbonylation determination

Protein carbonylation was measured using an OxiselectTM protein carbonyl ELISA Kit (Cell Biolabs Inc, San Diego, CA, USA) according to the instructions of the vendor. Briefly, 100 μL protein samples (10 μg/mL) were loaded onto the 96-well protein binding plate and incubated at 4 °C overnight. After washing three times, 100 μL dinitrophenylhydrazine (DNPH) working solution was added to react with the protein samples at room temperature for 45 min in the dark. After washing, 200 μL blocking buffer was added to the plate and incubated for 2 h at room temperature on a shaker. Thereafter, the plate was washed and incubated with 100 μL of anti-DNP for 1 h at room temperature. After washing three times, diluted HRP-conjugated secondary antibody was then added and incubated for another 1 h at room temperature followed by incubation with substrate solution for 10 min. Finally, absorbance of each sample was measured on a microplate reader and calculated using a protein carbonylation ELISA standard curve. The relative protein carbonylation level was expressed as a percentage of the sham group.

### Proteomic analysis of cytokine expression

A Proteome Profiler Rat Cytokine Array kit (R&D Systems, Minneapolis, MN, USA) was used to measure the levels of inflammatory cytokines in rat cortex and hippocampus following the manufacturer's instructions and the description in our previous study 27. In brief, the array's membranes were blocked using the Array Buffer 6 (block buffer) provided in the kit for 1 h. While the membranes were blocking, cortical or hippocampal samples from five animals in each experimental condition were pooled and 800 μg protein from each group was mixed with a reconstituted detection antibody cocktail and incubated for 1 h at room temperature. Thereafter, the blocked membranes were incubated with the prepared sample mixture overnight at 2-8 °C on a rocking platform shaker. The following day, after washing three times, the membrane was incubated with streptavidin-HRP for 30 min at room temperature. Finally, Chemi Reagent Mix was evenly added onto each membrane, and images were taken using the chemiluminescence system used in the Western blotting analysis. The calculated average density of each pair of dots was analyzed with ImageJ software (Version 1.49, NIH). The acquired values were transformed into z-scores, and a representative heat map was constructed using R package (R i386 3.6.2. lik). Log2 fold change was calculated.

### Myeloid cell and astrocyte morphology analysis

Immunofluorescence staining for Iba-1 (a myeloid cell marker) and GFAP (an astrocyte marker) was performed as described above. Confocal images were acquired as z-stacks with step sizes of 0.5 μm using a 63X objective lens on a LSM700 Meta confocal laser scanning microscope (Carl Zeiss). The images were viewed and reconstructed using Imaris software (Bitplane AG, Zürich, Switzerland). The smoothing was set at 0.4 μm for all channels and images. An appropriate threshold was set to differentiate the target signal from background noise and non-specific signals were removed. Astrocytes with a cell volume over 200 μm^3^ were selected for analysis. Typical images were selected for presentation.

Iba-1 immunostaining images were converted into representative skeletonized images using ImageJ plugins and analyzed by the software plugin AnalyzeSkeleton (2D/3D) following the instructions described in a previous study 50.

### Statistical Analysis

Statistical analyses were performed using ANY-maze (Stoelting) and SigmaStat software (Systat Software, San Jose, CA, USA). One-way analysis of variance (ANOVA) tests with Student-Newman-Keuls (S-N-K) post hoc tests were performed to determine differences between groups. All dependent variables with multiple time points or two interaction factors were analyzed using two-way repeated measures of ANOVAs followed by Tukey's all pairwise comparisons test to determine group difference. Data were expressed as mean ± SEM. *P* < 0.05 was considered significant for all statistical tests.

## Results

### Prenatal PBM improves survival rate and decreases infarct size after HI insult

To determine the effects of prenatal PBM treatment on neonatal HI rats, survival rate and brain infarct size were analyzed using a Kaplan-Meier survival curve and TTC staining, respectively. As shown in **Figure 1B**, all sham animals without HI insult survived a period of 2 h. In the HI group, 17 of 25 male animals (68%) survived for 2 h. For female animals, 17 of 24 animals (70.8 %) survived. Notably, prenatal PBM stimulation improved the 2-hour survival rate to 94.1% in both males and females, suggesting that prenatal PBM stimulation can protect against HI insult, regardless of sex. Regarding survival rate, no significant differences between females and males were detected.

TTC staining showed that the infarcted regions in HI rat brains were located primarily in the cortex and hippocampus (**Figure 1C**). HI insult generated an obvious infarcted area on brain sections taken from neonatal HI rats that was notably reduced in HI animals treated prenatally with PBM (**Figure 1C**). There are no significant differences regarding infarct size between females and males.

### Prenatal PBM alleviates cortex-related behavioral deficits after HI insult

As the infarct area in HI animals was located in the cortex and hippocampus, a battery of behavioral tests was performed to determine motor function. As shown in **Figure S1**, **Figure S2 and Figure S3**, no significant differences were detected between the untreated sham (sham) and the PBM-treated sham group (PBM+sham) in the behavioral tests and molecular level; therefore, the PBM-treated Sham group was not further examined.

**Figure 2:**
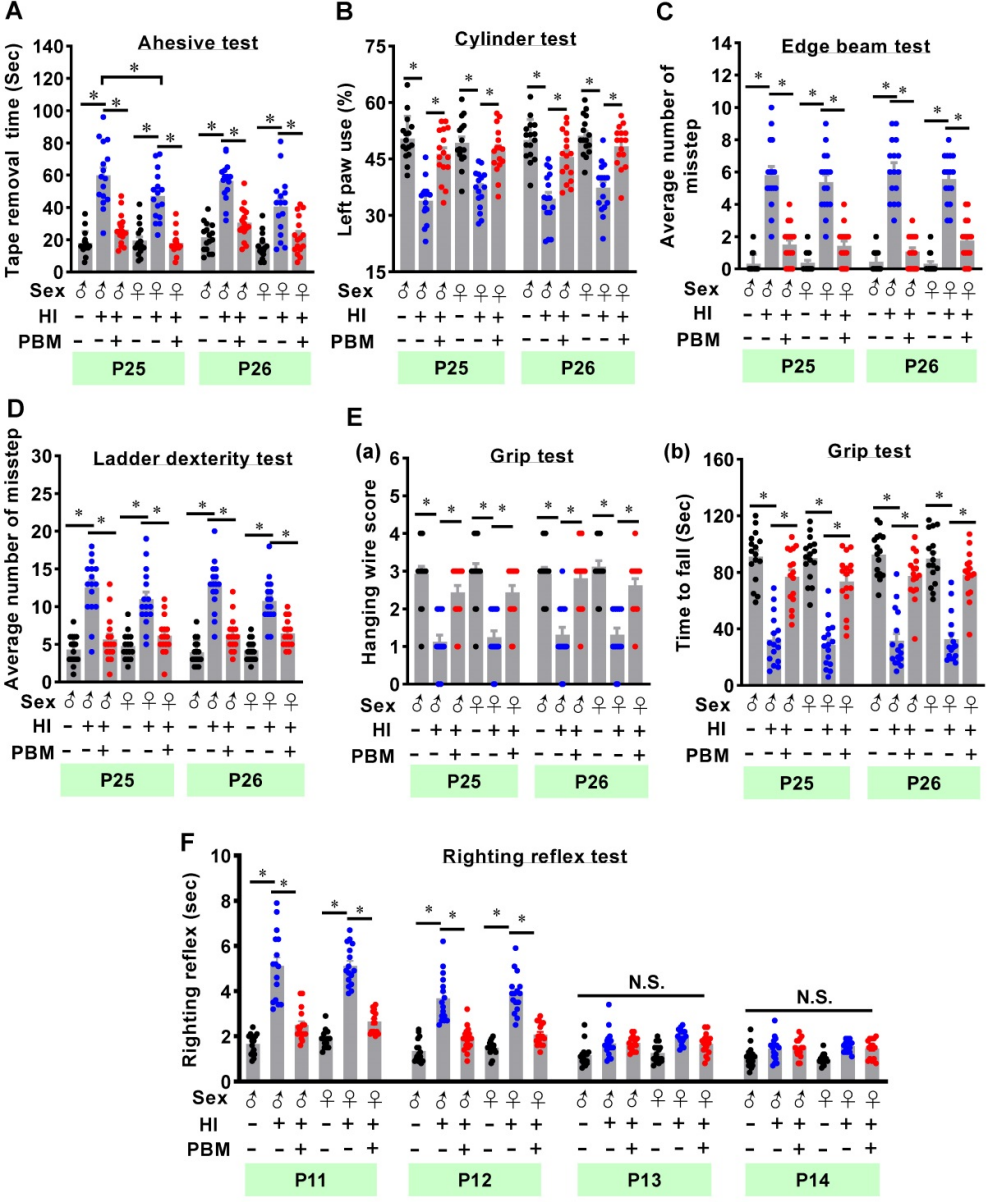
** Prenatal PBM alleviates cortex-related behavioral deficits after HI insult. (A)** The adhesive test measured somatosensory deficits induced by HI insult. **(B)** The cylinder test was performed to evaluate forelimb use. **(C)** The edge beam test was used to assess motor balance and coordination. **(D)** The ladder dexterity test, which investigated motor coordination, showed a decreased average number of missteps in PBM-treated HI rats compared to untreated HI rats. **(E)** The grip test analyzed forelimb motor coordination and grip strength. **(F)** The righting reflex test assessed basic motor coordination at early development ages (i.e., P11-P14) by measuring the time required to roll from back to paws. All data are expressed as mean ± SEM (n = 15-16). * indicates *P* < 0.05, “N.S.” indicates no significant difference (*P* > 0.05). ♂ indicates male; ♀ indicates females.

The adhesive test was performed on two consecutive days to measure the changes of forepaw somatosensory activity 26. As shown in **Figure 2A**, HI animals without prenatal PBM treatment spent significantly longer time removing the adhesive tape compared to sham animals. However, prenatal PBM treatment significantly reduced removal time, indicating that prenatal PBM treatment significantly alleviated somatosensory deficits induced by HI insult. In addition, male animals spent significantly longer time to remove tape compared with female HI animals at P25, indicating HI-induced somatosensory deficits in males were worse than females at P25. Additionally, the cylinder test was carried out to measure lateralized motor deficits. As shown in **Figure 2B**, animals in the HI group displayed significantly decreased use of the paw contralateral to the damaged hemisphere (i.e., the left paw) compared to sham animals. In contrast, prenatal PBM treatment significantly attenuated this deficit. Furthermore, results from the edge beam test and the ladder dexterity test showed that HI animals had significantly more missteps than sham animals, and notably, the number of missteps of PBM-treated HI rats was significantly reduced (**Figure 2C-D**). However, the completion time spent in the beam-walking test was not significantly different between the three groups (**Figure S4**). Grip test results revealed that prenatal PBM stimulation significantly improved forelimb strength, as evidenced by a significantly improved hanging wire score and an increased time to fall (**Figure 2E**). Finally, as noted in **Figure 2F**, HI animals displayed a significantly longer righting reflex time from P11 to P12 compared to sham animals, indicating HI injury delayed righting reflex development. Intriguingly, prenatal PBM treatment significantly attenuated this deficit on P11 and P12. No significant differences between females and males were detected in the cylinder test, edge beam test, ladder dexterity, grip test, and righting reflex.

### Prenatal PBM alleviates hippocampus-related behavioral deficits after HI insult

**Figure 3:**
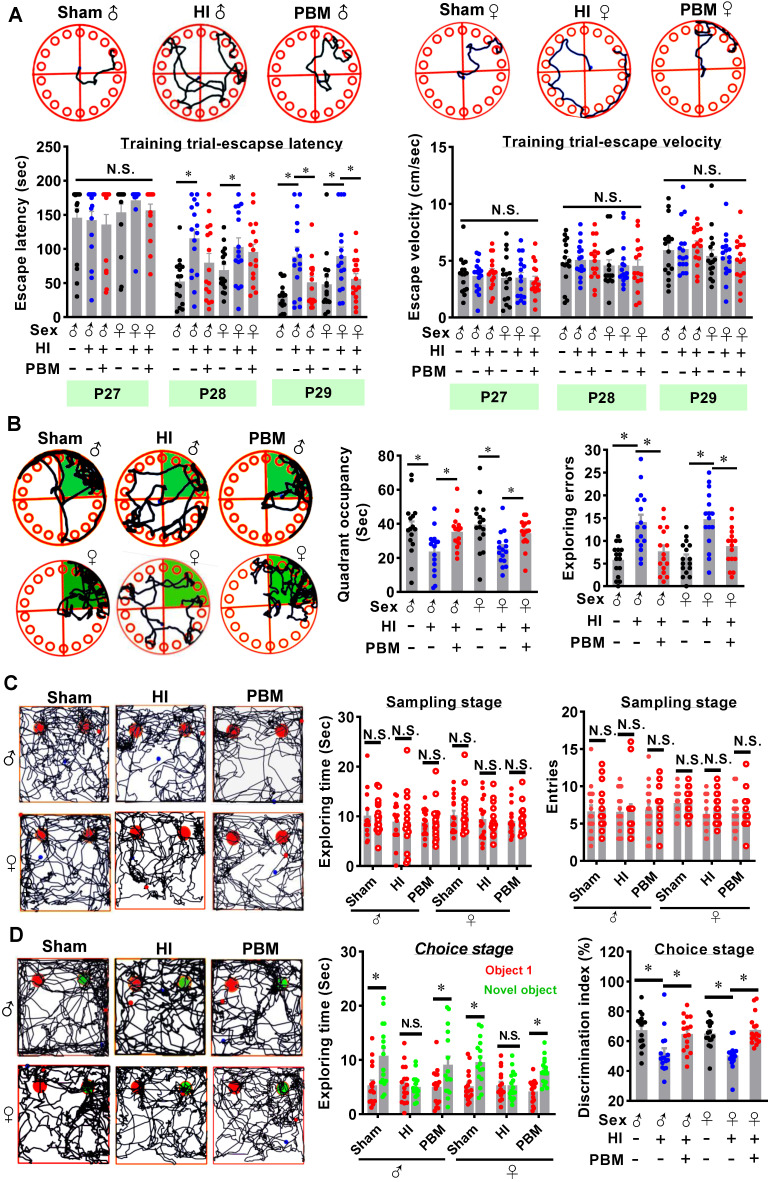
** Prenatal PBM alleviates hippocampus-related behavioral deficits after HI insult. (A)** The Barnes maze test was performed to measure hippocampus-dependent spatial learning and memory. Tracking plots, escape latencies, and average velocities were recorded from P27 to P29. **(B)** On P30, probe trials of the Barnes maze test were conducted. PBM-treated HI rats spent significantly more time in the target quadrant (green area) and made fewer exploring errors than their untreated counterparts. **(C)** Novel object recognition tests were performed to evaluate recognition memory. Exploring time of the two identical objects and entries to the area where the objects were located did not differ between the groups. **(D)** On the choice day, representative traces of animals exploring the familiar object (red) and the novel object (green) were recorded. HI rats spent significantly less time exploring the novel object (i.e., lower discrimination index) than either the sham rats or the HI rats treated prenatally with PBM. All data are presented as mean ± SEM (n = 15). * indicates *P* < 0.05, “N.S.” indicates no significant difference (*P* > 0.05). ♂ indicates male; ♀ indicates females.

The Barnes maze is a widely accepted test measuring hippocampus-dependent spatial learning and memory 46. Rats were subjected to training trials from P27 to P29 and probe trials on P30. As shown in **Figure 3A**, animals that underwent HI insult spent a longer time finding the hidden box (i.e., increased escape latency) on P28 and P29 compared to sham rats. In contrast, the escape latency was significantly decreased on P29 in the prenatal PBM treatment group (**Figure 3A**). Notably, the escape velocity was not significantly different between groups (**Figure 3A**), which was consistent with the results in edge beam test (**Figure S4**), indicating that the difference in escape latency was not due to a variation in velocity. In addition, during the probe trials (**Figure 3B**), HI rats had a significant decrease in occupancy time in the quadrant where the escape box had been located, suggesting remarkable cognitive deficits. Interestingly, PBM-treated HI neonates spent significantly longer time in the target quadrant compared to untreated HI rats (**Figure 3B**). Furthermore, HI animals presented significantly increased exploration errors on probe day compared to sham animals, and this effect was significantly attenuated in the PBM-treated HI animals (**Figure 3B**).

To determine whether prenatal PBM treatment could preserve recognition memory after HI insult, novel object recognition tests were performed. As shown in **Figure 3C**, on the sampling stage, animals in all three groups did not show a significant difference in preference for the two identical objects. Notably, on the choice day, animals from the sham group and the PBM group spent more time exploring the novel object than animals from the HI group (**Figure 3D**), suggesting that recognition memory in HI animals was impaired and that recognition memory was preserved by prenatal PBM stimulation. There were no significant differences between sexes in these hippocampus-related behavioral deficits.

### Prenatal PBM attenuates synaptic, dendritic, and white matter injury

**Figure 4:**
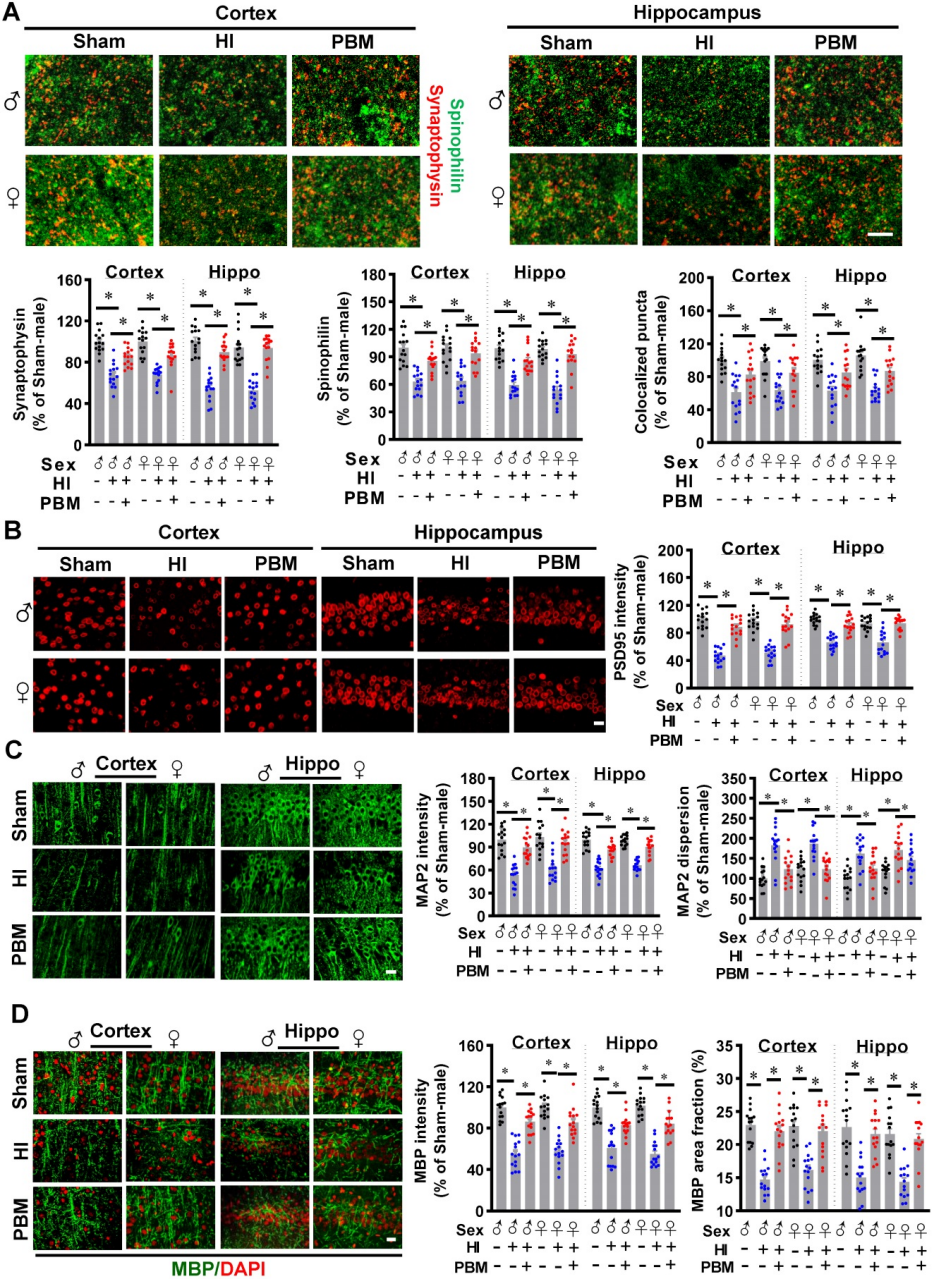
** Prenatal PBM attenuates synaptic, dendritic, and white matter injury. (A)** The granule density of the presynaptic marker synaptophysin (red) and the dendritic spine marker spinophilin (green) was significantly reduced in both the cortex and hippocampus of HI rats as compared to sham and PBM-treated HI rats. Colocalization of these two markers was also reduced. **(B)** The density of postsynaptic marker PSD-95 was significantly reduced in the cortex and hippocampus of HI rats compared to sham and PBM-treated HI rats. **(C)** Expression of the dendritic marker MAP2 was significantly attenuated in the cortex and hippocampus of HI animals compared to sham and PBM-treated HI animals. However, the dispersion of MAP2 expression increased. **(D)** The fluorescent intensity and area fraction of MBP, a white matter marker, significantly decreased in the cortex and hippocampus of HI rats when compared to sham and PBM-treated HI rats. Scale bar = 10 µm. All data are presented as mean ± SEM (n = 15). * indicates *P* < 0.05. ♂ indicates male; ♀ indicates females.

HI insult in neonatal rats results in synaptic, dendritic and white matter injury 51-53. Therefore, we next investigated the effects of prenatal PBM treatment on the morphology of synapses and dendrites and on the expression of myelin basic protein (MBP), a marker for white matter injury. As shown in **Figure 4A**, expression of the presynaptic marker synaptophysin and the dendritic spine marker spinophilin in the cortex and hippocampus showed that HI insult significantly decreased the number of synaptic granules and the expression of spinophilin. Prenatal PBM treatment significantly attenuated this decrease, suggesting that prenatal PBM treatment significantly alleviated HI-induced synaptic loss and reduced spinophilin expression. In addition, the PBM-treated HI group exhibited an increased number of colocalized synaptic puncta compared to the untreated HI group (**Figure 4A**), suggesting that PBM significantly protected synaptic structure from HI insult. As shown in **Figure 4B**, expression of postsynaptic marker PSD95 in the cortex and hippocampus confirmed the effect of prenatal PBM on synaptic structure.

MAP2 is a widely used dendritic marker and a very sensitive marker for assessing neuronal damage 1. The results depicted in **Figure 4C** show that HI rats displayed decreased MAP2 intensity and increased MAP2 dispersion in both the cortex and hippocampus compared to sham animals. Prenatal PBM treatment alleviated this effect.

Furthermore, myelin basic protein (MBP), an indispensable protein of myelinated axons, is a sensitive predictive biomarker of white matter injury 54, 55. As shown in **Figure 4D**, HI rats evidenced a lower MBP fluorescent intensity and MBP area fraction compared to sham animals. In contrast, PBM treatment of HI animals attenuated this decrease, suggesting that prenatal PBM stimulation protects against white matter injury and axonal damage. No significant differences between females and males were detected.

### Prenatal PBM represses HI-induced neuronal degeneration and apoptosis

**Figure 5:**
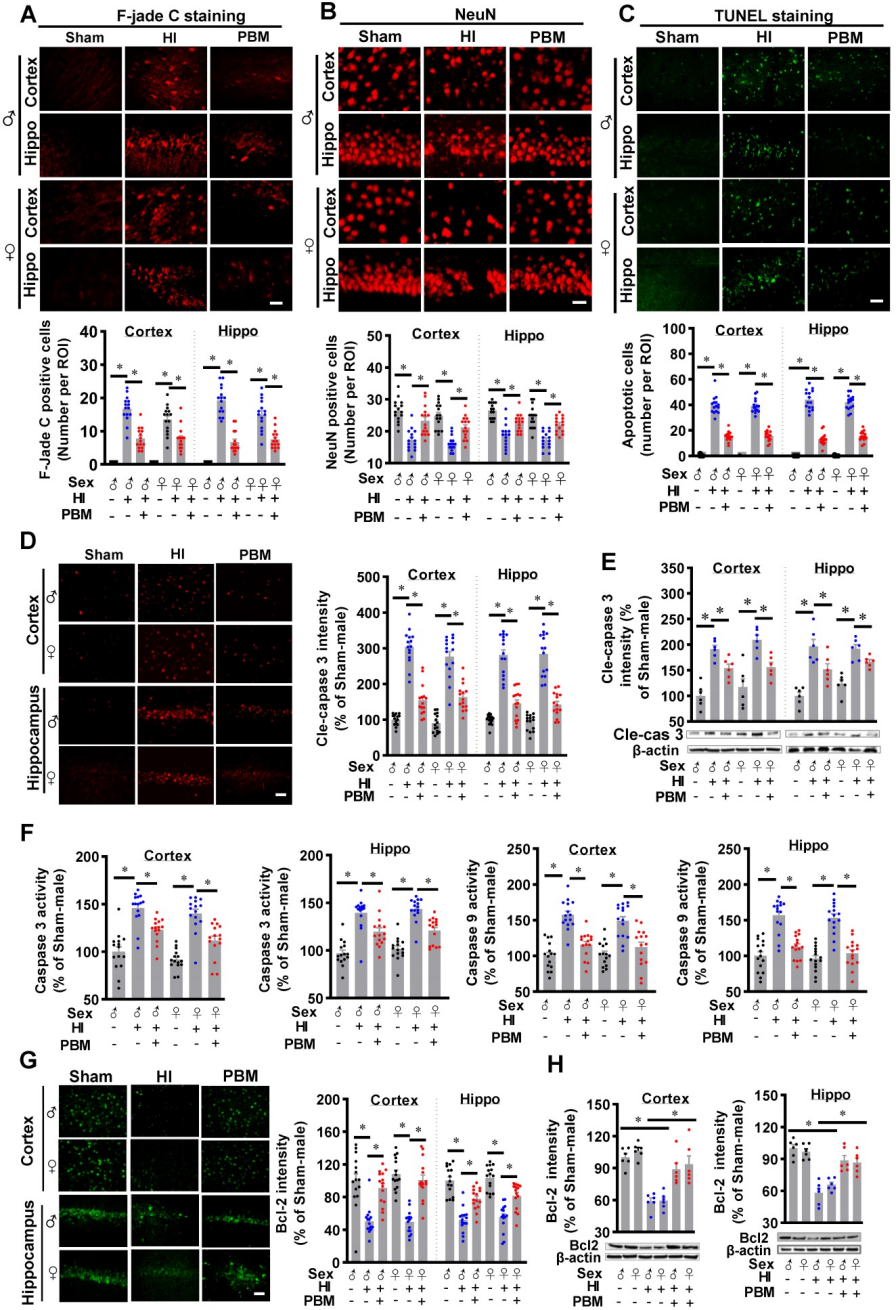
** Prenatal PBM represses HI-induced neuronal degeneration and apoptosis. (A)** The number of F-Jade C positive cells in the cortex and hippocampus. **(B)** The number of NeuN positive cells in the cortex and hippocampus. **(C)** The number of apoptotic cells as quantified by TUNEL staining. **(D)** Levels of cleaved-caspase 3 were increased in the cortex and hippocampus. **(E)** Western blot results of cleaved-caspase 3 in the cortex and hippocampus. **(F)** Caspase 3 and caspase 9 activities were measured using cytosolic proteins. Both proteins were more highly expressed in HI animals than sham or PBM-treated HI animals. **(G)** The density of Bcl2 in the cortex and hippocampus. **(H)** Western blot results of Bcl2 in the cortex and hippocampus. All data are presented as mean ± SEM (n = 6-15). Scale bar = 20 µm. * indicates *P* < 0.05. ♂ indicates male; ♀ indicates females.

We next investigated the efficacy of prenatal PBM treatment on HI-induced neuronal degeneration and apoptosis. Typical Fluoro-Jade C staining showed that HI insult significantly increased Fluoro-Jade C positive cells in the cortex and hippocampus, suggesting neuronal degeneration significantly increased in the HI group (**Figure 5A**). This effect was markedly reduced by prenatal PBM treatment. In addition, as shown in **Figure 5B**, confocal microscopy revealed a significant loss of NeuN positive cells in both the cortex and hippocampus of HI animals compared to the sham group. In contrast, neuronal loss was significantly attenuated by prenatal PBM treatment. Consistent with these results, as shown in **Figure 5C**, HI insult resulted in a remarkably increased number of TUNEL-positive cells compared with sham animals and PBM-treated animals, suggesting that prenatal PBM stimulation significantly repressed HI-induced apoptosis.

Subsequently, immunostaining for the active form of caspase 3 was performed. A predominant activation of caspase 3 occurred in the cortex and hippocampus of HI rats, but caspase 3 activation was significantly attenuated by PBM treatment (**Figure 5D**). In addition, western blot results in **Figure 5E** confirmed this result. Furthermore, the activity of caspase 3 and caspase 9 were measured using protein samples. As shown in **Figure 5F**, both caspase 3 and caspase 9 activity in the cortex and hippocampus were significantly increased following HI insult. In contrast, this increase was repressed by prenatal PBM treatment. Immunostaining for Bcl-2, a regulator of the intrinsic apoptotic pathway 56, provided additional support for the anti-apoptotic efficacy of PBM in the context of HI insult, as evidenced by the decreased expression of Bcl-2 following HI insult and preserved expression in the PBM-treated HI group (**Figure 5G-H**). No significant differences between females and males were detected.

### Prenatal PBM preserves cytochrome *c* oxidase activity and ATP production after HI insult

**Figure 6:**
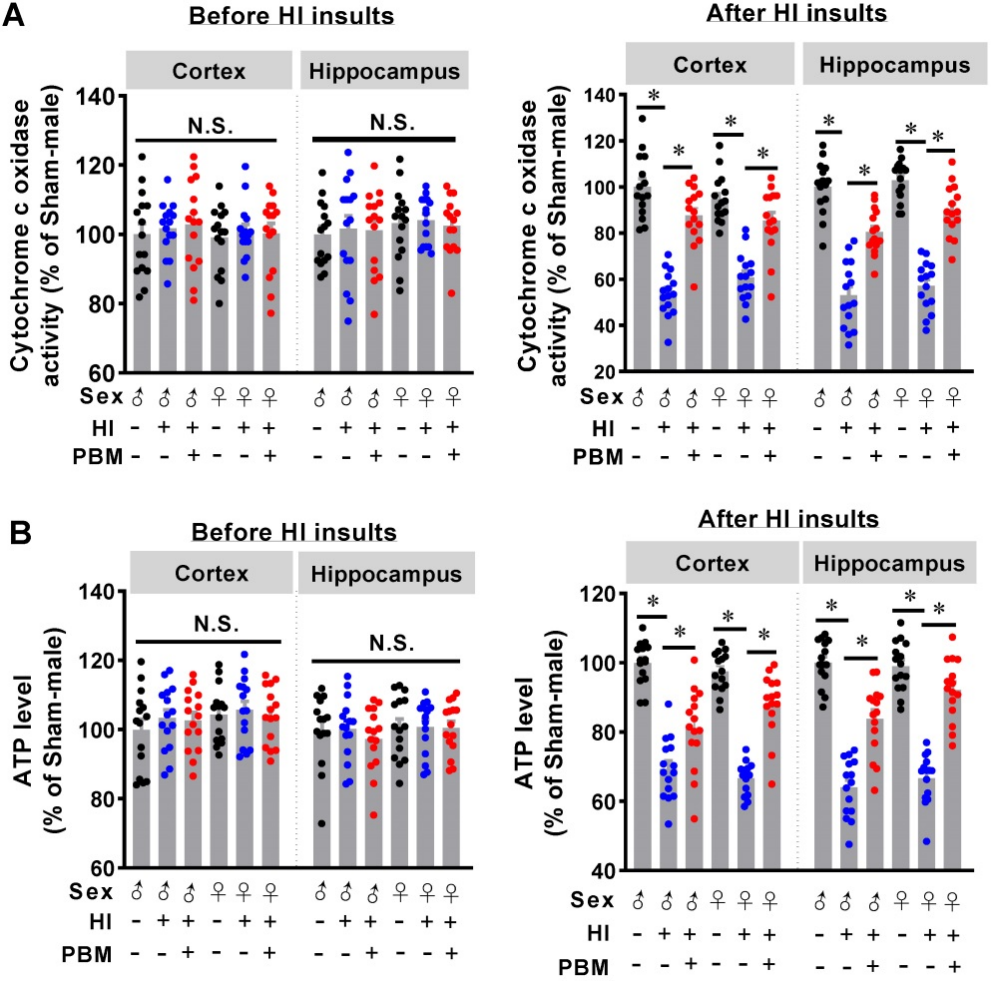
**Prenatal PBM preserves mitochondrial cytochrome *c* oxidase activity and ATP production. (A)** Mitochondrial cytochrome *c* oxidase (CCO) activity in the cortex and hippocampus was measured before and after HI insult. There was no difference in activity between groups prior to HI insult, but CCO activity decreased in rats after HI insult. However, prenatal PBM treatment protected against this decrease. **(B)** ATP content in the cortex and hippocampus before and after HI insult was measured and followed a similar pattern. All data are presented as mean ± SEM (n = 15). * indicates *P* < 0.05; “N.S.” indicates not significant difference (*P* > 0.05). ♂ indicates male; ♀ indicates females.

Cytochrome *c* oxidase (CCO) is one of the main targets of PBM 28. Therefore, we next investigated whether prenatal PBM treatment could affect CCO activity. As shown in **Figure 6A**, we did not find significant differences in CCO activity between the three groups before the P10 HI insult. After HI insult on P10, however, significantly decreased CCO activity was observed in HI animals. Interestingly, prenatal PBM treatment significantly restored CCO activity (**Figure 6A**).

To further investigate the effects of prenatal PBM treatment on mitochondrial function, ATP content in the cortex and hippocampus was measured. As shown in **Figure 6B**, there were no differences in ATP content between the three groups before HI insult, suggesting that prenatal PBM stimulation did not change the ATP production in offspring normal animals at P10. However, prenatal PBM treatment significantly improved ATP levels after HI insult (**Figure 6B**), which is consistent with the CCO activity results. Taken together, these results indicate that prenatal PBM treatment did not affect normal mitochondrial function and CCO activity. However, prenatal PBM treatment significantly improved mitochondrial resistance to HI insult. No significant differences between females and males were detected.

### Prenatal PBM protects mitochondrial dynamics and mitochondrial membrane potential

Mitochondria are majorly affected organelles in HI injury 14, and mitochondrial dynamics and mitochondrial membrane potential play a key role in the maintenance of CCO activity and ATP production 57. Therefore, we next investigated the effects of prenatal PBM therapy on HI-induced changes in mitochondrial dynamics and mitochondrial membrane potential.

**Figure 7:**
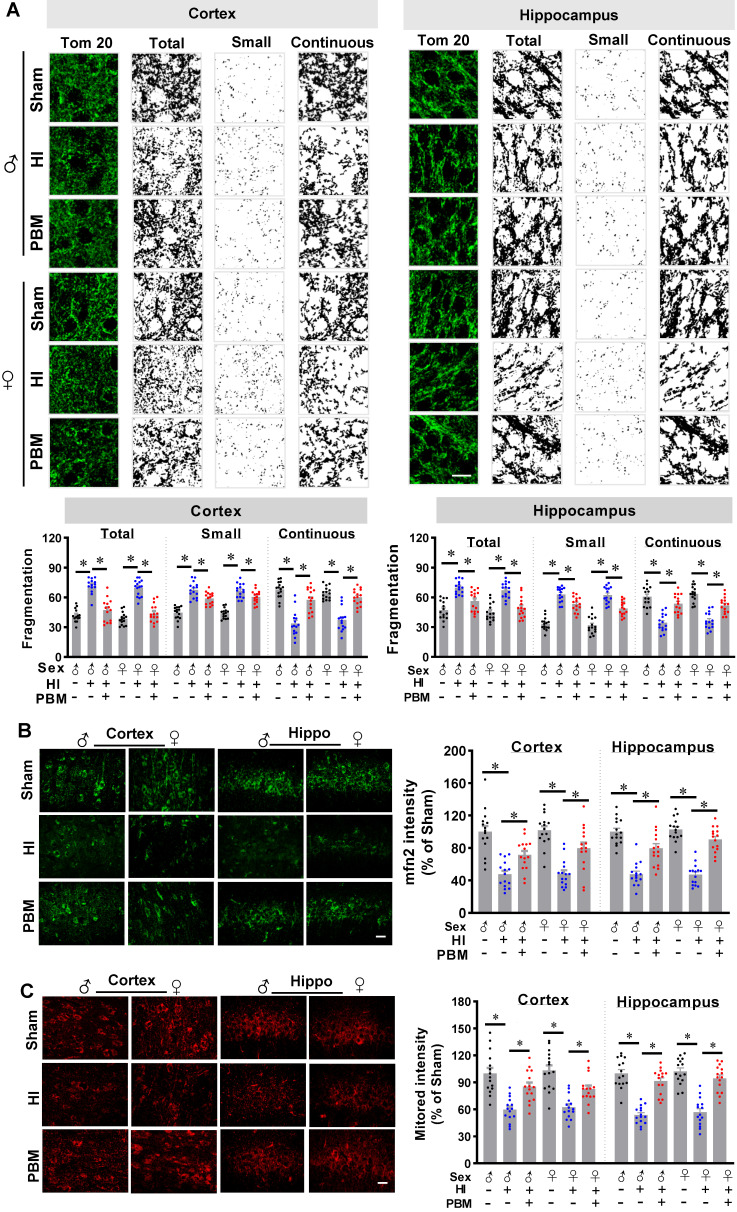
**Prenatal PBM preserves mitochondrial dynamics and mitochondrial membrane potential. (A)** Confocal microscopy of Tom20, a mitochondrial outer membrane marker, showed increased mitochondrial fragmentation in the cortex and the hippocampus of HI animals and attenuated fragmentation in PBM-treated HI animals. Following image thresholding, filtering (median, 2.0 pixels), binarization, and analysis with Image J, mitochondrial segments were separated as total particles, small particles (size < 1.5 µm), and continuous structures (size > 2 µm). The number of total particles and small particles were normalized using total mitochondrial area. Continuous structures were expressed as the percentage of the area of large particles normalized to the total mitochondrial area. **(B)** Confocal staining for the mitochondrial fusion protein Mfn2 evidenced decreased expression in the cortex and hippocampus of HI animals and restored expression in HI animals treated prenatally with PBM**. (C)** The mitochondrial membrane potential was measured using MitoTracker Red. The fluorescence intensity was qualified and normalized as a percentage change compared to the sham group. Scale bar = 10 µm. All data are presented as mean ± SEM (n = 15). * indicates *P* < 0.05. ♂ indicates male; ♀ indicates females.

As shown in **Figure 7A**, staining for the outer mitochondrial membrane marker Tom20 was used to visualize mitochondria in the cortex and hippocampus 1. Of significant interest, we found that total mitochondrial fragmentation and the number of small particles (size < 1.5 μm) were significantly increased following HI insult compared with the sham group. However, mitochondrial fragmentation was attenuated in the HI group treated prenatally with PBM, as was the number of small particles. Consistent with these results, the amount of continuous mitochondrial structure was decreased in the HI group and preserved in HI animals treated prenatally with PBM (**Figure 7A**).

Additional staining demonstrated a significantly decreased expression of Mfn2, one of the key effectors for mitochondrial fusion 58, in HI animals compared to sham animals. Of importance, Mfn2 expression was significantly increased in PBM-treated HI animals (**Figure 7B**).

Finally, mitochondrial membrane potential (MMP) was examined using MitoTracker Red fluorescent dye. As shown in **Figure 7C**, MitoTracker Red fluorescence in the cortex and hippocampus was significantly decreased following HI insult, suggesting HI-induced mitochondrial depolarization and potential collapse of the mitochondrial membrane. Notably, the decreased MitoTracker Red intensity was attenuated in PBM-treated HI rats, indicating a preservation of MMP in these animals. No significant differences between females and males were detected.

### Prenatal PBM inhibits mitochondrial dysfunction-induced oxidative stress

**Figure 8:**
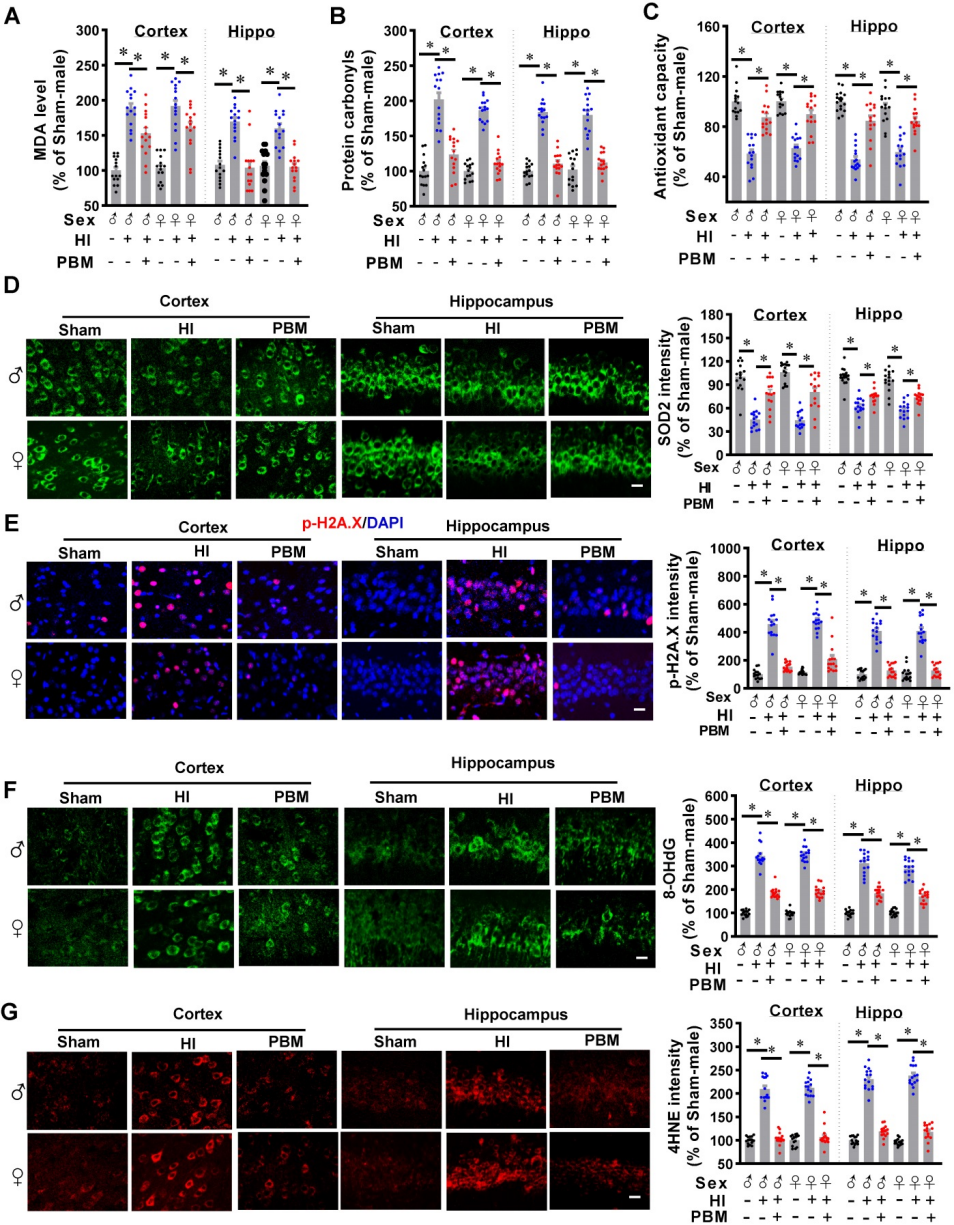
** Prenatal PBM inhibits HI-induced oxidative stress. (A)** MDA levels and **(B)** protein carbonyl levels increased while **(C)** total antioxidant capacity and **(D)** SOD2 expression decreased in the cortex and hippocampus of rats subjected to HI insult. Prenatal PBM treatment was able to ameliorate these changes. **(E)** DNA double-strand breaks were analyzed using p-H2A.X staining, **(F)** oxidized DNA damage was assessed using 8-OHdG staining, and **(G)** lipid peroxidation was measured using 4-hydroxynonenal (4-HNE) staining. The results of the HI group and the PBM-treated group are quantified as percentage changes versus the sham group. Scale bar = 10 µm. All data are presented as mean ± SEM (n = 15). * indicates *P* < 0.05. ♂ indicates male; ♀ indicates females.

Mitochondrial dysfunction also plays a central role in the formation of oxidative stress 28. Therefore, we next investigated the effects of prenatal PBM treatment on HI-induced oxidative stress. As shown in **Figure 8A**, HI injury induced a significant increase in MDA production in the cortex and hippocampus compared with the sham group. This increased production of MDA was attenuated by prenatal PBM treatment.

In addition, as shown in **Figure 8B**, we found that HI induced a significantly increase of protein carbonyls, an effect that could be alleviated by PBM treatment. Furthermore, total antioxidant capacity was determined using an antioxidant assay kit. **Figure 8C** indicated that the total antioxidant capacity was significantly decreased in HI rats compared with sham animals, and the total antioxidant capacity was preserved in animals with prenatal PBM treatment.

Furthermore, to measure the effects of prenatal PBM treatment on HI-induced oxidative stress, the expression of antioxidant enzyme (SOD2) and products of oxidative stress such as phosphor-H2AX (p-H2A.X), 8-hydroxy-2'-deoxyguanosine (8-OHdG), and 4-hydroxynonenal (4-HNE), were measured via immunostaining in the cortex and hippocampus. As illustrated in **Fig. 8D**, representative confocal microscopy images demonstrated a robust decrease in immunoreactivity for SOD2 in HI group compared to Sham, and a dramatic increase in PBM group animals compared to HI group without PBM treatment. In addition, HI insult significantly increased the levels of p-H2A.X, 8-OHdG, and 4-HNE within the cortex and hippocampus compared to sham animals (**Figure 8E-G**), indicating increased oxidative damage to proteins, DNA, and lipids. The levels of these oxidative stress products were significantly decreased in the cortex and hippocampus of rats treated with PBM prenatally. No significant differences between females and males were detected.

### Prenatal PBM reduces myeloid cells activation, promotes a shift in M1/M2 phenotype, and suppresses pro-inflammatory cytokine production

**Figure 9:**
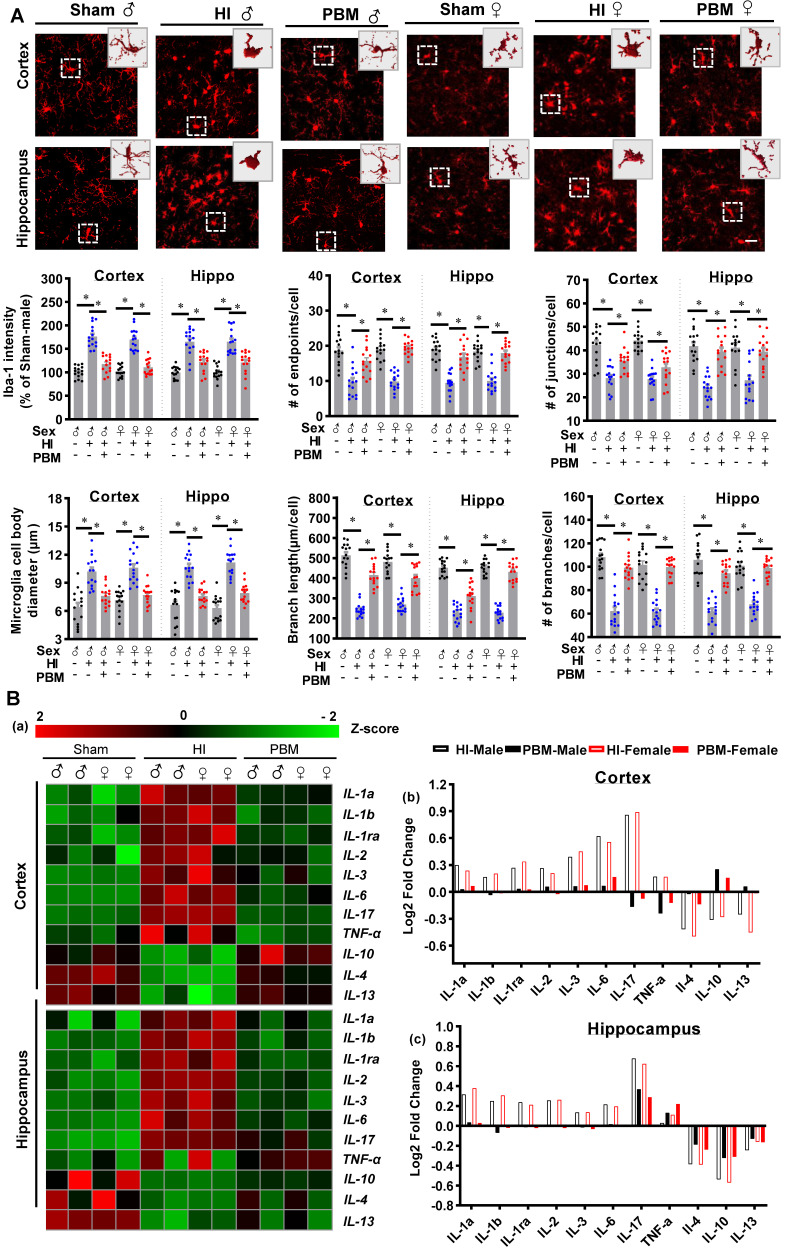
** Prenatal PBM reduces the excessive activation of macrophage and pro-inflammatory cytokine production. (A)** Macrophage were stained using Iba-1 antibody and the representative confocal images of Iba-1 were analyzed. In HI animals, Iba-1 intensity and cell body diameters increased while the number of junctions, number of endpoints, number of branches, and length of branches decreased compared to both sham and PBM-treated HI animals. Rectangles: cells enlarged and 3D-rendered. Scale bar = 20 µm. Data are presented as mean ± SEM (n = 15). **(B)** Compared to expression in sham rats, pro-inflammatory cytokine expression was shown to be higher in HI rats, while anti-inflammatory cytokine expression was lower. Prenatal PBM treatment of HI rats attenuated these effects. Data are Log2 fold change (cortical or hippocampal tissue from 5 animals were mixed for each sample). * indicates *P* < 0.05. ♂ indicates male; ♀ indicates females.

Myeloid cell over-activation is known to affect local inflammation and contribute to neuronal damage 26, 27. As shown in **Figure 9A**, expression of Iba-1 increased in HI animals and significantly decreased in PBM-treated HI animals. Furthermore, myeloid cell morphology was analyzed using ImageJ software (Version 1.49, NIH). Results showed significantly increased myeloid cell body diameter; a decreased number of junctions, endpoints, and branches; and decreased branch length after HI insult, suggesting that myeloid cell were activated after HI insult. In contrast, myeloid cell in the PBM group had longer branches and smaller body sizes compared to the HI group, indicating those myeloid cells were at a relatively quiescent state.

Therefore, we investigated the effects of prenatal PBM therapy on inflammatory cytokine expression and myeloid cell polarization. To better observe the changes of inflammatory cytokine levels in the cortex and hippocampus, the acquired values were transformed into z-scores, and a representative heat map was constructed using R software. As shown in **Figure 9B**, the levels of 8 pro-inflammatory cytokines (i.e., IL1-α, IL-1β, IL-1ra, IL-2, IL-3, IL-6, IL-17, and TNF-α) were markedly elevated in the cortex and hippocampus of HI rats. In contrast, expression of these pro-inflammatory cytokines was suppressed in the PBM group compared to the HI group while the expression of the anti-inflammatory cytokines IL-4, IL-10, and IL-13 increased in the PBM-treated HI group relative to the untreated HI group (**Figure 9B**).

**Figure 10:**
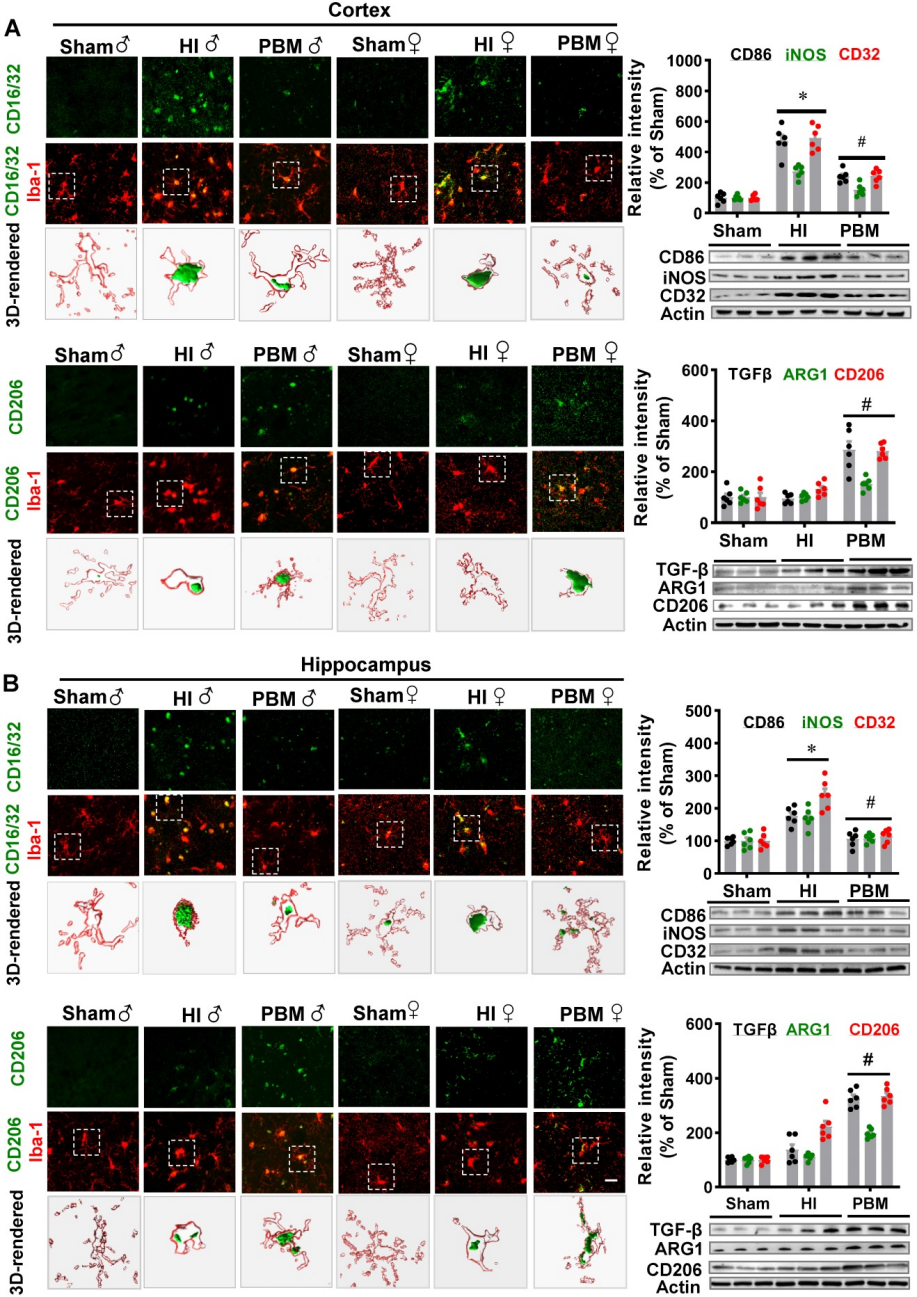
** Prenatal PBM shifts microglial polarization from M1 to M2 phenotype. (A)** Double-label immunofluorescence of microglial marker Iba-1 with the M1 marker CD16/32 or the M2 phenotype marker CD206 in the cortex showed an increase in CD16/32^+^Iba1^+^ cells in HI rats relative to sham. Prenatal PBM treatment of HI rats attenuated this increase while increasing the number of CD206^+^/Iba1^+^ cells relative to both sham and HI rats. **(B)** The effects of PBM on M1 and M2 markers in the hippocampus followed the same trend. Rectangles: cells enlarged and 3D-rendered in the bottom row. Scale bar = 20 µm. Results of western blot analysis showed an increased expression of the M1 markers CD86, iNOS, and CD32 in HI male rats compared to sham. PBM treatment decreased the expression of M1 markers and increased the expression of the M2 markers, TGFβ, ARG1, and CD206. All data are expressed as mean ± SEM (n = 6). * indicates *P* < 0.05. ♂ indicates male; ♀ indicates females.

To determine whether prenatal PBM treatment could affect a shift between M1 and M2 phenotypes, brain slices were stained using double-label immunostaining for the myeloid cell marker Iba-1 and either the inflammatory M1 phenotype marker CD16/32 or the anti-inflammatory M2 phenotype marker CD206. As shown in **Figure 10A-B**, we observed a marked increase in CD16/32^+^Iba1^+^ cells in the cortex of HI animals compared with the sham group. In contrast, the number of CD16/32^+^Iba1^+^ cells were reduced in the cortex of HI animals with prenatal PBM treatment. We also observed an increased number of CD206^+^/Iba1^+^ cells in the cortex of animals treated prenatally with PBM. Furthermore, western blotting analysis of M1 markers in the male animals revealed significantly increased levels of M1 markers (i.e., CD32, CD86, and iNOS) in the HI group and significantly decreased levels in the PBM group (PBM vs. HI). Interestingly, M2 markers (i.e., ARG1, TGFβ, and CD206) were significantly elevated in the PBM-treated versus non-treated HI group, suggesting that prenatal PBM treatment facilitates M2 anti-inflammatory phenotype polarization.

### Prenatal PBM attenuates neurotoxic A1 astrocyte activation after HI insult in favor of a neuroprotective A2 phenotype

**Figure 11:**
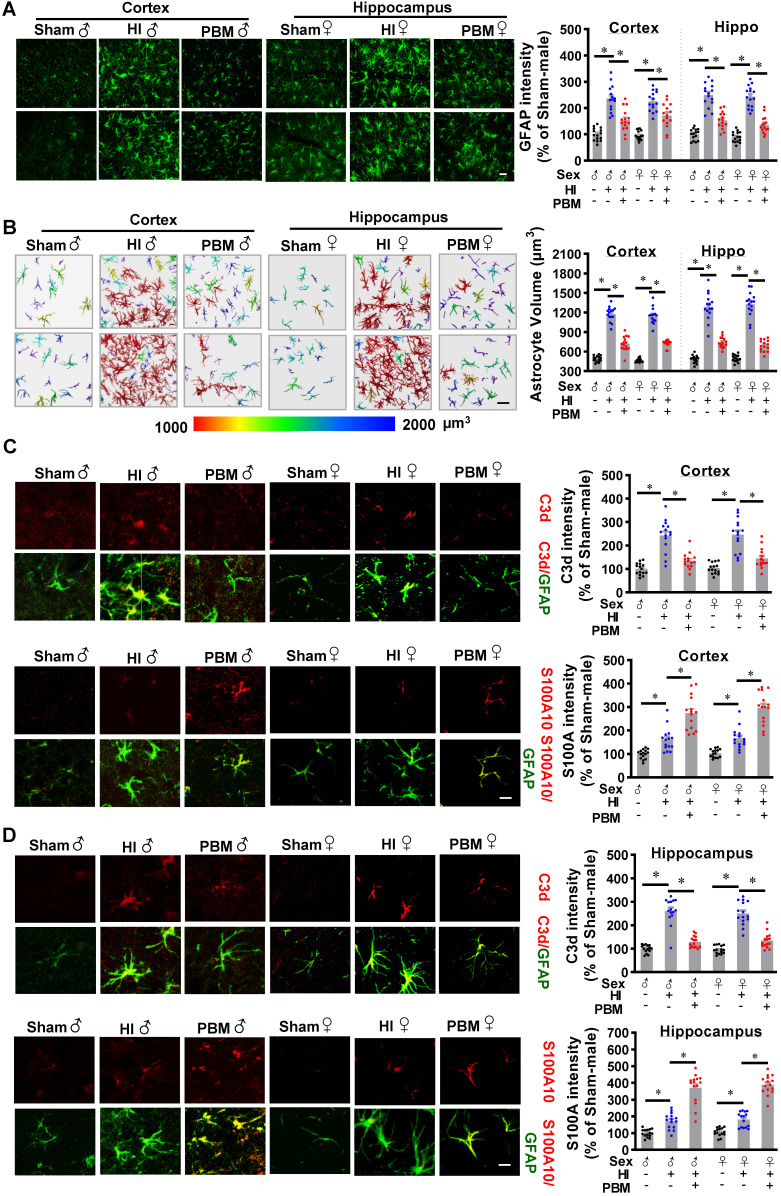
** Prenatal PBM attenuates neurotoxic A1 astrocyte activation after HI insult in favor of a neuroprotective A2 phenotype. (A)** Astrocyte activation was measured using the astrocyte marker GFAP. GFAP fluorescent intensity was analyzed using Image J software and expressed as a percentage change versus sham. **(B)** Representative 3D images are shown of astrocytes in the cortex and hippocampus with cell body volumes distinguished using different colors. Astrocyte volumes from the cortex and hippocampus were measured and quantitatively analyzed. Typical double-label immunofluorescence of GFAP with C3d (an A1 phenotype marker) or S100A10 (an A2 phenotype marker) in the cortex **(C)** and hippocampus **(D)** showed that PBM attenuated an HI-induced increase in A1 astrocytes and augmented an HI-induced increase in A2 astrocytes**.** Scale bar = 10 µm. All data are presented as mean ± SEM (n = 15). * indicates *P* < 0.05. ♂ indicates male; ♀ indicates females.

A previous study reported that neurotoxic reactive astrocytes can be activated by microglia, a type of myeloid cells. Therefore, we next investigated the effects of prenatal PBM stimulation on astrocyte activation and a switch between A1 and A2 phenotypes. As shown in **Figure 11A**, GFAP immunoreactivity increased in the HI group compared with the sham group, but this effect was attenuated by PBM treatment. In addition, we found that astrocytes in the HI group were larger compared to those in the sham group, indicating a robust activation of astrocytes after HI insult. In contrast, the astrocytes in the PBM-treated group appeared remarkably less hypertrophic compared to the astrocytes from the HI group.

To further analyze and quantitate cell volume, we constructed 3D images of astrocytes in the cortex and hippocampus using Imaris software. The results (**Figure 11B**) revealed that astrocyte volume was significantly increased in HI animals compared to sham animals. In contrast, astrocyte volume was significantly lower in PBM-treated versus non-treated HI rats. These observations suggest that prenatal PBM treatment impairs the induction of reactive astrocytes after HI insult.

Double-immunostaining was performed to detect both the neurotoxic A1 (C3d) and the neuroprotective A2 (S100A10) phenotypes of astrocytes 27. As shown in **Figure 11C-D**, C3d was strongly expressed and co-localized with GFAP in the cortex and hippocampus of HI animals. Examination of PBM-treated HI rats revealed a significant reduction in C3d expression as compared to untreated HI animals. In contrast, HI insult induced mild but significant increases in S100A10 expression, which were further increased by PBM pretreatment, suggesting that PBM treatment improves the brain's ability to activate the neuroprotective A2 astrocyte phenotype. Taken together these results indicate that prenatal PBM stimulation is capable of attenuating A1 astrocyte activation and is able to induce an A1 to A2 phenotype shift. No significant differences between females and males were detected.

## Discussion

Our findings support the possible use of prenatal PBM treatment to prevent HI-induced brain injury in pregnancies at high risk of HIE. The current study supplied evidence that prenatal PBM treatment reduces HI-associated mortality rate in neonatal rats and also exerts protective properties against brain tissue loss and functional deficits. According to a previous study, neonatal HI injury induces behavioral deficits via synaptic dysfunction, neuronal structural damage, and neuronal loss 1, 2, 59. In the present study, we demonstrated that prenatal PBM treatment significantly attenuates synaptic, dendritic, and white matter damage. Administered as a pre-treatment, the neuroprotective effect of prenatal PBM treatment is, at least in part, due to the following mechanisms: (1) improvement of mitochondrial homeostasis and dynamics and maintenance of the mitochondrial membrane; (2) reduction in oxidative stress and damage; (3) suppression of excessive myeloid cell activation via a shift from an M1 pro-inflammatory phenotype to an M2 anti-inflammatory phenotype; and (4) attenuated activation of a neurotoxic A1 astrocyte phenotype in favor of a neuroprotective A2 astrocyte phenotype.

Currently, the only licensed treatment option for neonatal HIE is therapeutic hypothermia 60, 61. The underlying mechanism for therapeutic hypothermia is complex and includes reduction of cerebral metabolism and the energy requirement of neurons; suppression of oxygen free radical release and lipid peroxidation; attenuation of apoptotic processes; suppression of myeloid cell activation; and induction of pro-inflammatory cytokine release 62-64. Although therapeutic hypothermia significantly improves outcomes 62-64, the limited time period for effective application requires that hypothermia must be administered within 6 h of birth; such a small window of opportunity may be difficult to accommodate if the equipment is not readily available or an HIE diagnosis is made late 1, 11, 65. Therapeutic hypothermia administered 6-24 h after delivery may be beneficial, but its effectiveness is uncertain 8. Many patients still suffer severe brain damage and disability 60, 66. Therefore, a more effective and practical method is desperately needed.

In the past few decades, PBM treatment has shown beneficial effects on multiple brain disorders in animal models and in limited human studies including ischemic stroke 67, 68, Parkinson's disease 69, Alzheimer's disease 30, depression 70, and anxiety 71. According to previous studies, laser wavelengths in the red (i.e., 600-700 nm) and near-infrared (NIR, 760-1200 nm) range are the most commonly used wavelengths for PBM medical applications 26, 72, 73. Lasers with a wavelength of 808 nm have demonstrated deepest tissue penetration and, as a result, have been widely used for various brain disorders 1, 2, 26, 47, 74, 75. Numerous cells/tissues responded to PBM, including neurons 76, microglia 77, astrocytes 78, embryonic rat brains 79, mouse embryonic stem cells 80, and chicken embryos 81. Furthermore, 808 nm low-level laser light penetrated the rabbit skull and brain to a depth of 2.5-3 cm when placed upon the skin surface in previous reports 82-84. In the current study, the laser energy was able to propagate through the abdominal tissue of the dam and the brain of the neonate received a power density of 8 mW/cm^2^. In addition, according to the results of a previous study, this level of laser irradiation did not induce temperature changes or tissue damage at the applied dose 26, 85.

Pretreatment with PBM has been attracting increasing attention in the field of preconditioning 86. Previous studies have used preconditioning regiments consisting of hypothermia, physical exercise, ischemia, and hyperthermia to improve resistance to future ischemic insult 87, 88. Preconditioning activates pathways resistant to ischemic insult and causes cells to shift to protective cellular phenotypes 89-92. This present study conducted prenatal PBM treatment to demonstrate the effects of PBM preconditioning on neonatal animals undergoing HI insult. In the current study, we chose to perform HIE model on P10 rat pups, as the P9 or P10 mouse brain has been recognized as the developmental equivalent of a full-term neonatal human brain, rather than P7, according to a recent study 42. As reported in a previous study 93, placing P7 pups in a hypoxic environment with 8% oxygen induced morbidity at rates upwards of 20%. In the current study, P10 animals were placed in a hypoxic environment with 6% oxygen which induced a higher morbidity. PBM treatment, however, significantly improved the survival rate. Impaired motor coordination, retarded performance in righting reflexes, and memory and learning deficits have been found in both human and animal neonates after HI injury 1, 3, 32, 94. In the current study, we found prenatal PBM treatment was able to attenuate HI-induced impaired motor coordination, retarded righting reflexes, and memory and learning deficits, while causing no significant differences (i.e., adverse side effects) in healthy animals. In addition, prenatal administration of PBM alleviated HI-induced neurodegeneration and neuronal apoptosis as well as synaptic, dendritic, and white matter damage. In contrast with post-treatment, prenatal PBM treatment in the current work was performed as a pretreatment. Therefore, the decreased infarct area in the PBM treatment may be mediated by the improvement of mitochondria function, decreased inflammation, and reduced levels of oxidative damage.

It is well-known that HI insult in the newborn has profound molecular consequences which stem from energy failure 95. Therefore, the beneficial effects of prenatal PBM treatment on behavioral improvement and neuronal protection may be related to mitochondria, the primary target of PBM treatment 1, 2. The effects of PBM on mitochondrial morphogenesis, mitochondrial membrane potential, and ATP synthesis have been reported by numerous studies 79, 96-99. The most well-studied mechanism of PBM treatment is its ability to enhance ATP production by increasing CCO activity 68. Previous studies, including our own, have demonstrated that PBM application on the brain can increase CCO activity and increase ATP production in different animal models 1, 26, 31, 47, 79, 100. In the current study, before HI insult, there were no differences in ATP content or CCO activity in the cortex and hippocampus of rat subjects. According to a previous study, within 12 h of PBM application, ATP levels in the neonatal brain decreased to normal levels 1. In the current study, ATP content in the brain of neonatal rats was measured at least 10 days after prenatal PBM treatment, well after the ATP levels of treated mice would have returned to normal levels according to previous reports 1, 101. Interestingly, our results showed that ATP content and CCO activity in both the cortex and hippocampus were significantly decreased after HI insult. However, the ATP levels and CCO activity in the PBM-treated HI group were well-preserved, indicating that prenatal PBM treatment improved mitochondrial resilience to HI insult. This conclusion was further supported when we showed that HI-induced mitochondrial fragmentation and mitochondrial membrane potential disruption were significantly attenuated by prenatal PBM treatment.

Oxidative stress and neuroinflammation has been recognized as closely related pathophysiological processes in HIE 102, 103. It is well-known that, following an HI insult, mitochondrial dysfunction results in the production of excess ROS, which rapidly overwhelms neuronal antioxidant capacity 16. The high levels of ROS induce oxidative damage to cellular macromolecules, which initiates a cycle of ROS production and organelle dysregulation that eventually triggers neuronal apoptosis 1, 16, 104-107. We found that prenatal PBM treatment significantly alleviated HI-induced oxidative damage to lipids, proteins, and DNA. Taken together, we demonstrated that prenatal PBM application significantly attenuated excess ROS production and thereby improved the resistance of neurons to HI insult.

In neonatal HIE in particular, overexpression of inflammatory cytokines has been associated with brain damage and poor prognosis 108-110. Furthermore, myeloid cell activation after neonatal HIE has been well-documented 111, 112. The M1 pro-inflammatory macrophage phenotype is associated with increased production of pro-inflammatory cytokines 113-116, and the M2 anti-inflammatory macrophage phenotype is related to tissue repair, phagocytosis of protein aggregates, and removal of cellular debris 117, 118. An M1/M2 polarization has been shown to play an important role in HIE pathophysiological development 119. As a result, myeloid cell activation and pro-inflammatory cytokine release have become well-accepted targets for neonatal HIE therapies 119-121. In the current study, we demonstrated that prenatal PBM treatment has an inhibitory effect on myeloid cell activation and pro-inflammatory cytokine release. Furthermore, we found that prenatal PBM treatment promotes an M1 to M2 phenotype conversion.

In addition to myeloid cell activation, accumulating evidence suggests that excess astrocyte activation has negative effects on normal restorative processes after brain injury 122-124. Preventing astrogliosis in the neonatal brain has been shown to improve neurogenesis after neonatal HI 122, 125. Similarly to myeloid cell, astrocytes can also be classified into an A1 neurotoxic phenotype or an A2 neuroprotective phenotype 27, 126. The A1 neurotoxic phenotype can secrete cytokines that induce neuronal damage, whereas the A2 neuroprotective phenotype is able to enhance neuronal survival 126. Therefore, therapies that induce a shift from the A1 phenotype to the A2 phenotype may improve HI patient outcomes. In our study, we found that prenatal PBM treatment significantly inhibited astrogliosis and promoted a shift towards the A2 neuroprotective phenotype. Taken together, our results demonstrate that prenatal PBM treatment is not only able to prevent astrocytes from causing damage but is also able to induce astrocytes to perform beneficial functions.

In the current study, although we found beneficial effects of prenatal PBM treatment on rat offspring undergoing neonatal hypoxic ischemia, more work must be undertaken to discern the underlying mechanisms, optimal dosages, and possible side effects of such treatment before it can be implemented in the clinic. In addition, according to previous studies, HIF-α dimerizes with HIF-β in hypoxic conditions, as detailed in previous studies 98, 127, 128. PBM, however, could induce this affect further and increase HIF levels, indicating that HIF signaling is a valuable line of future investigation. If prenatal PBM is proven effective in humans, people with pregnancies at high risk of HIE may benefit from prenatal PBM treatment, as maternal cigarette smoking, malnutrition, and fetal exposure to nicotine, alcohol, cocaine and glucocorticoids may directly or indirectly act at cellular and molecular levels to alter brain development and result in heightened brain vulnerability to hypoxic-ischemic encephalopathy and the development of neurological diseases in postnatal life 129-134. For these people, PBM treatment may be a potential approach to protect their baby against damage caused by neonatal HI insult. This merits in-depth future investigation including both animal and preclinical studies. In addition, future work is still needed on investigating the treatment using PBM during or after HIE induction.

**Figure 12:**
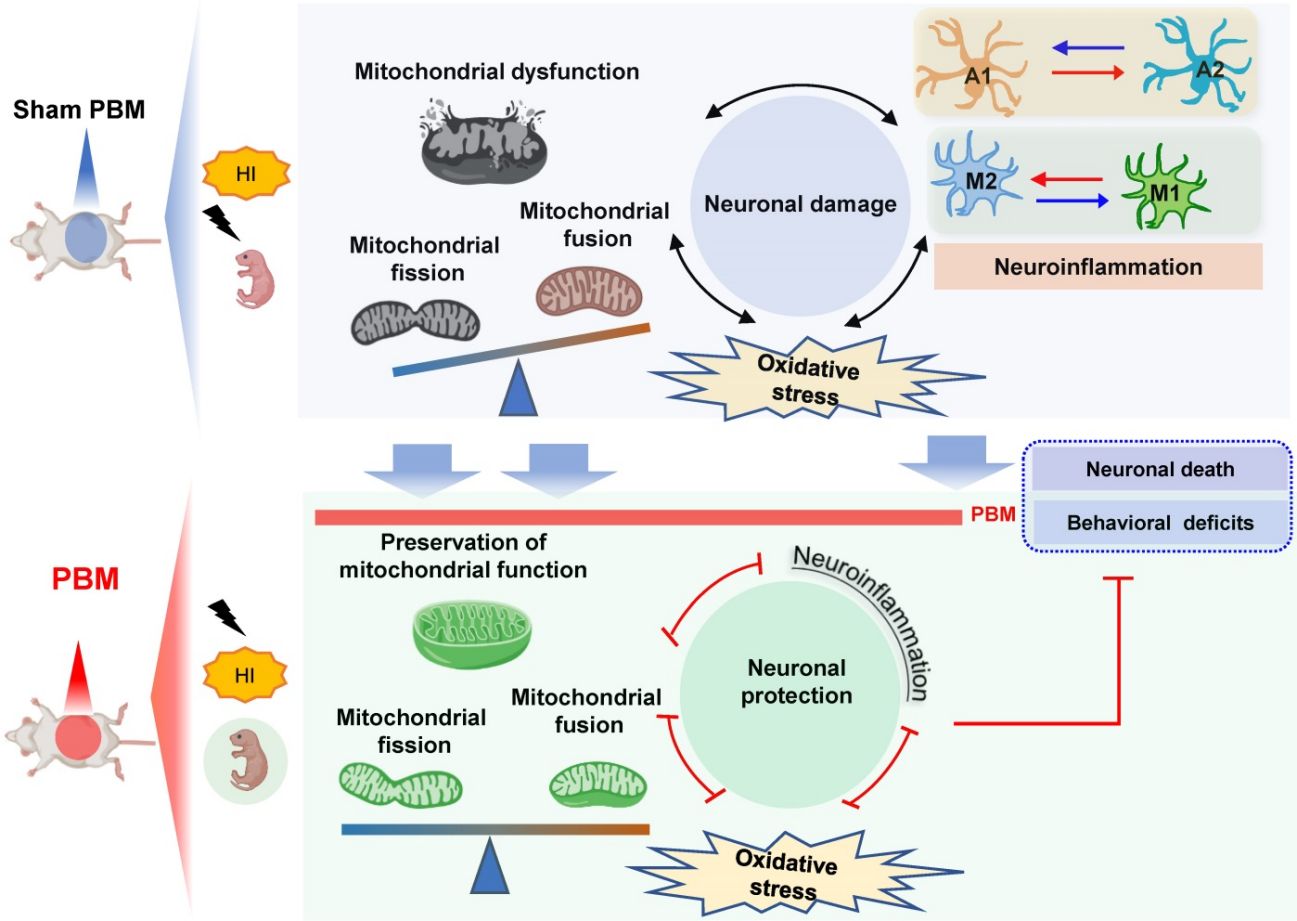
** Prenatal PBM protects against HI-induced neurodegeneration and behavioral deficits.** Prenatal PBM treatment protects against damage caused by HI insult in several ways. PBM treatment attenuates HI-induced mitochondrial dysfunction; decreases oxidative stress; suppresses neuroinflammation; reduces astrogliosis and excessive activation of macrophage; and promotes a shift toward anti-inflammatory macrophage phenotypes and neuroprotective astrocyte phenotypes. These combined mechanisms result in enhanced survival rates, reduced neurodegeneration, and improved behavioral outcomes.

In conclusion, our study demonstrates that pretreating rats with PBM during the prenatal period can protect these animals from later damage caused by neonatal HI insult. The results of our study also suggest that the mechanism underlying the benefits of prenatal PBM treatment includes the preservation of mitochondrial function and mitochondrial integrity; inhibition of neuroinflammation and oxidative stress; reduction of excessive microgliosis and astrogliosis; and regulation of the glial polarizing status (**Figure 12**).

## Supplementary Material

Supplementary figures and tables.

## Abbreviations

4-HNE: 4-hydroxynonenal
8-OHdG: 8-hydroxy-2'-deoxyguanosine
BSA: bovine serum albumin
CCO: cytochrome *c* oxidase
DHE: Dihydroethidium
DNPH: dinitrophenylhydrazine
GD0: gestation day 0
HI: hypoxic-ischemic
HIE: Neonatal hypoxic-ischemic encephalopathy
MMP: mitochondrial membrane potential
NIR: near-infrared
NOR: novel object recognition
OD: optical density
P10: postnatal day 10
PBM: Photobiomodulation
p-H2A.X: phosphor-H2AX
PVDF: polyvinylidene difluoride
PVDF: polyvinylidene difluoride
RECCA: right common carotid artery
ROS: reactive oxygen species
SOD2: superoxide dismutase 2
MBP: myelin basic protein
