# Supplementary material for: Effects of prenatal photobiomodulation treatment on neonatal hypoxic ischemia in rat offspring
Source: Theranostics. 2021 Jan 1;11(3):1269–94. doi: 10.7150/thno.49672 (PMC7738878; doi:10.7150/thno.49672)
Supplement: Supplementary file 1 — Supplementary figures and tables. [file thnov11p1269s1.pdf]

Supplementary Material

Supplementary Figures and Figure Legends

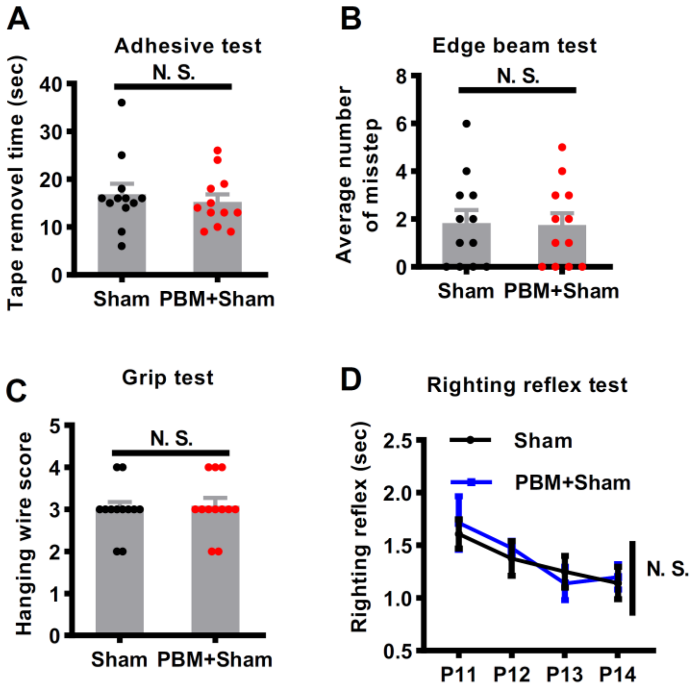

Supplemental Figure S1. Prenatal PBM did not affect motor function in normal rats. Results for the **(A)** adhesive test, **(B)** edge beam test, **(C)** grip test, and **(D)** righting reflex test were not significantly different between sham groups with/without PBM (Sham and PBM+Sham groups). All data are presented as mean ± SEM (n = 12). "N.S." indicates no significant difference ($P > 0.05$).

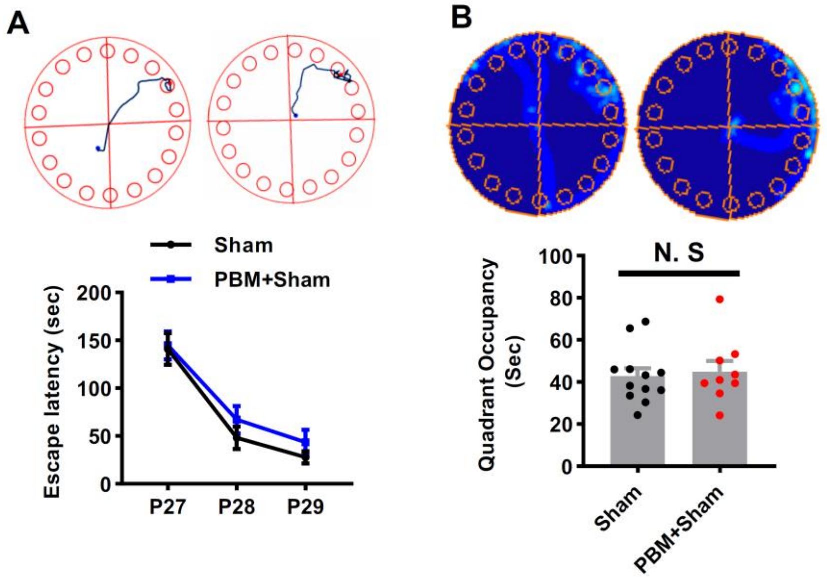

Supplemental Figure S2. Prenatal PBM did not affect learning and memory in normal rats. (A) Results for escape latency and **(B)** quadrant occupancy were not significantly different between sham groups with/without PBM treatment (Sham and PBM+Sham groups). All data are presented as mean ± SEM (n = 9-12). “N.S.” indicates not significant ($P > 0.05$).

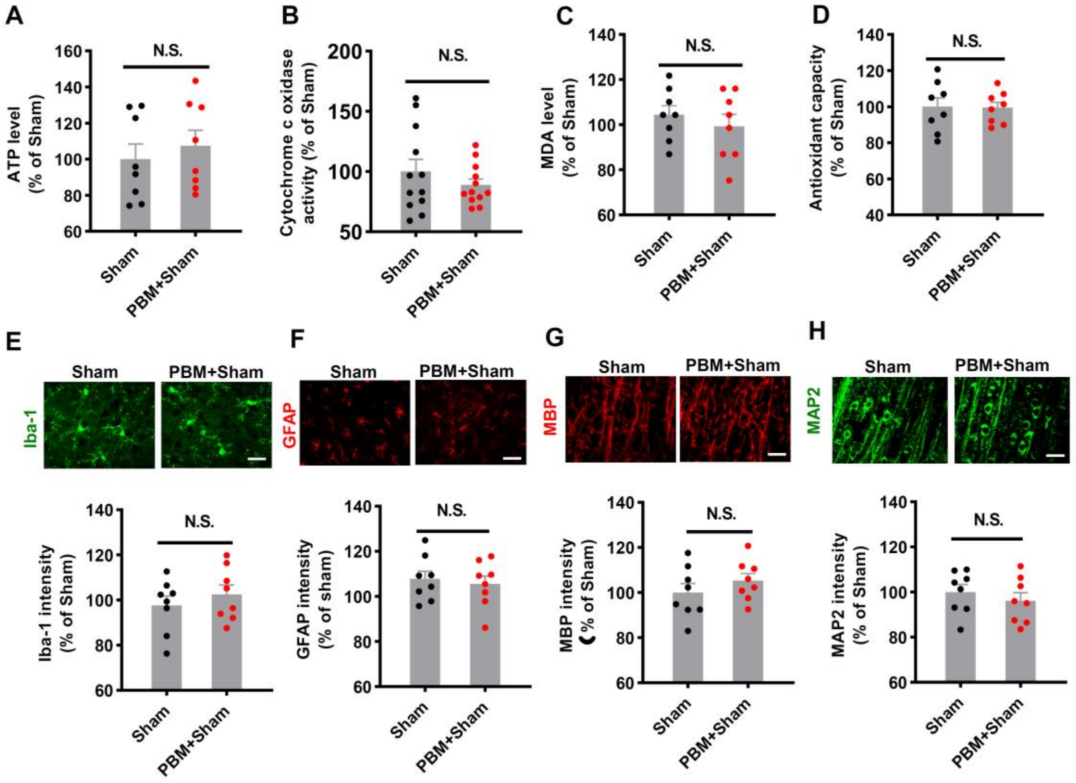

Supplemental Figure S3. No differences were found in molecular level between Sham and PBM+Sham group. Results for the **(A)** ATP content, **(B)** CCO activity, **(C)** MDA level, **(D)** total antioxidant capacity, **(E)** the intensity of Iba-1, **(F)** GFAP, **(G)** MBP and **(H)** MAP2 were not significantly different between sham groups with/without PBM (Sham and PBM+Sham groups). Scale bar = 20 μm. All data are presented as mean ± SEM (n = 8). "N.S." indicates no significant difference ($P > 0.05$).

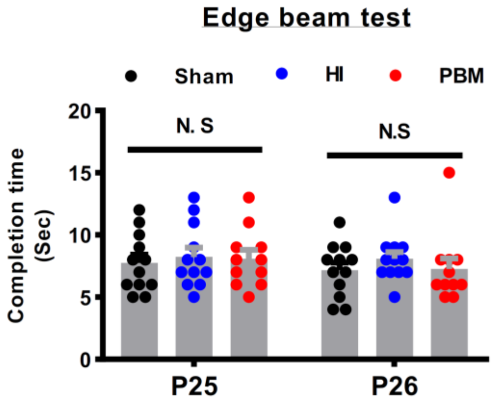

Supplemental Figure S4. HI insult and prenatal PBM do not affect velocity. The edge beam test indicated no significant difference in escape velocity between the three groups. All data are presented as mean ± SEM (n = 12). "N.S." indicates not significant ($P > 0.05$).

Table S1

Device information

Manufacturer	Changchun new industries Optelectronics Tech, CP., L
Model identifier	MDL-III-808-500 mW
Number of emitters	1
Wavelength	808 ± 3.0
Spatial distribution of emitters	Round LED spotlight
Emitter type	Diode IR laser
Beam delivery system	Fiberoptic

Supplemental Table S1. Device information for the Diode IR Laser System used for prenatal PBM treatment.
